# Palladium Nanoparticles Hardwired in Carbon Nanoreactors Enable Continually Increasing Electrocatalytic Activity During the Hydrogen Evolution Reaction

**DOI:** 10.1002/cssc.202101236

**Published:** 2021-09-15

**Authors:** Mehtap Aygün, Melanie Guillen‐Soler, Jose M. Vila‐Fungueiriño, Abdullah Kurtoglu, Thomas W. Chamberlain, Andrei N. Khlobystov, Maria del Carmen Gimenez‐Lopez

**Affiliations:** ^1^ Centro Singular de Investigación en Química Biolóxica e Materiais Moleculares (CIQUS) Universidade de Santiago de Compostela 15782 Santiago de Compostela Spain; ^2^ School of Chemistry University of Nottingham University Park Nottingham NG7 2RD United Kingdom; ^3^ Institute of Process Research and Development School of Chemistry University of Leeds Leeds LS2 9JT United Kingdom; ^4^ Nanoscale & Microscale Research Centre University of Nottingham University Park Nottingham NG7 2RD United Kingdom; ^5^ Present address: Faculty of Science Erzurum Technical University Erzurum 25050 Turkey

**Keywords:** carbon nanoreactors, electrocatalysis, hydrogen production, palladium nanoparticles, sustainable chemistry

## Abstract

Catalysts typically lose effectiveness during operation, with much effort invested in stabilising active metal centres to prolong their functional lifetime for as long as possible. In this study palladium nanoparticles (PdNP) supported inside hollow graphitised carbon nanofibers (GNF), designated as PdNP@GNF, opposed this trend. PdNP@GNF exhibited continuously increasing activity over 30000 reaction cycles when used as an electrocatalyst in the hydrogen evolution reaction (HER). The activity of PdNP@GNF, expressed as the exchange current density, was always higher than activated carbon (Pd/C), and after 10000 cycles PdNP@GNF surpassed the activity of platinum on carbon (Pt/C). The extraordinary durability and self‐improving behaviour of PdNP@GNF was solely related the unique nature of the location of the palladium nanoparticles, that is, at the graphitic step‐edges within the GNF. Transmission electron microscopy imaging combined with spectroscopic analysis revealed an orchestrated series of reactions occurring at the graphitic step‐edges during electrocatalytic cycling, in which some of the curved graphitic surfaces opened up to form a stack of graphene layers bonding directly with Pd atoms through Pd−C bonds. This resulted in the active metal centres becoming effectively hardwired into the electrically conducting nanoreactors (GNF), enabling facile charge transport to/from the catalytic centres resulting in the dramatic self‐improving characteristics of the electrocatalyst.

## Graphical abstract

**Room for self‐improvement**: Palladium nanoparticles confined inside hollow graphitised carbon nanofibers exhibit a remarkable increasing activity over 30000 reaction cycles when used as electrocatalyst in the hydrogen evolution reaction, surpassing the activity of platinum on carbon. During operation, palladium becomes hardwired into the graphitic step‐edges that open, enabling facile charge transport to/from the catalytic centres resulting in the dramatic self‐improving characteristics of the electrocatalyst.

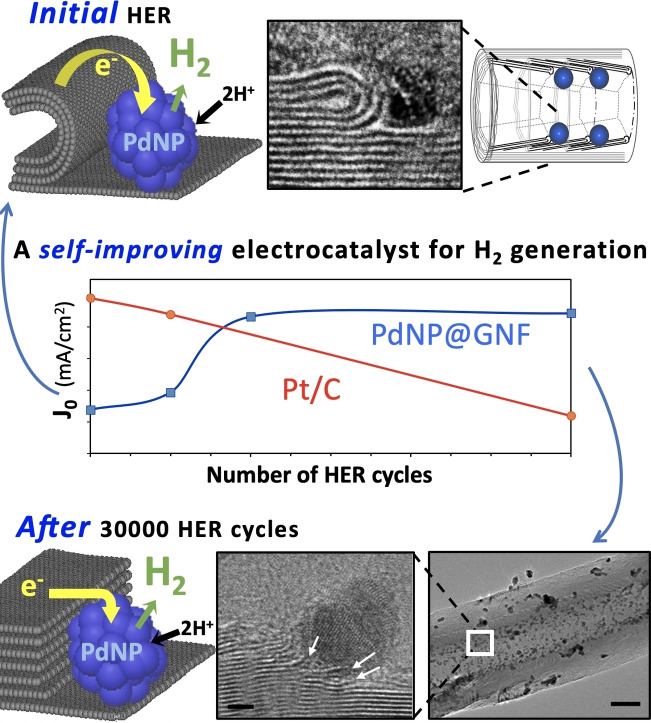

## Introduction

In the last two decades there has been an increasing demand for clean and renewable energy sources as alternatives to fossil fuels.[^1^, ^2^, ^3^, ^4^] For instance, the water‐splitting reaction, which consists of the hydrogen and oxygen evolution half‐reactions (HER and OER, respectively), has attracted a great deal of attention as a sustainable source of hydrogen (H_2_).[^5^, ^6^, ^7^] Hydrogen gas has been identified as a key energy carrier that can be used to produce clean electricity in fuel cells, where the hydrogen oxidation and oxygen reduction reactions (HOR and ORR, respectively) convert chemical energy into electrical energy.[^7^, ^8^] Furthermore, driving the HER with renewable sources of energy can lead to a sustainable source of hydrogen fuel that can be used in a zero‐emission fuel cell. The hydrogen evolution reaction (2H^+^+2e^−^→H_2_), the cathodic reaction in electrochemical water splitting, is a classic example of a two‐electron transfer reaction with one catalytic intermediate, H* (where * indicates a site on the electrode surface) and may occur through either the Volmer‐Heyrovsky or the Volmer‐Tafel mechanism [Eqs. (1)–3]:

(1) $$\mathrm{Volmer}\;\mathrm{step}:\;H{}^{+}+e{}^{-}+{}^{*}\to H{}^{*}$$

(2) $$\mathrm{Heyrovsky}\;\mathrm{step}:\;H{}^{*}+H{}^{+}+e{}^{-}\to H{}_{2}+{}^{*}$$

(3) $$\mathrm{Tafel}\;\mathrm{step}:\;2H{}^{*}\to H{}_{2}+2{}^{*}$$

Achieving high energy efficiency for water splitting requires the use of a catalyst to minimize the overpotential necessary to drive the HER. Platinum‐based catalysts are historically the best‐performing materials in the HER, owing to the negligible overpotential required to achieve high reaction rates in acidic solutions.[^7^, ^8^, ^9^, ^10^, ^11^, ^12^] However, their high cost, the global scarcity of Pt and the limited reusability of such materials have stimulated exploration of alternatives to Pt‐based electrocatalysts.[^11^, ^12^, ^13^, ^14^, ^15^, ^16^, ^17^, ^18^] Among those alternatives, transition metal‐based materials have received significant attention due to their high abundance and low price; however, their much higher overpotentials make them less effective electrocatalysts for HER. For example, Pd is more abundant than Pt and exhibits higher electrocatalytic activity and stability than other transition metal‐based materials and their alloys. As the use of platinum group metals in the catalytic converters of gasoline engines will be obsolete due to the arrival of electric cars, using Pd as an electrocatalyst could become a viable way forward. Recently, different synthetic strategies have been explored to design cost‐effective Pd‐based electrocatalysts aiming to increase the number of active sites and/or their intrinsic activity, including nanostructuring, bimetallic alloying and dispersion on carbon.[^17^, ^18^, ^19^, ^20^, ^21^] For example, modification of Pd with MoS_2_ (Pd−MoS_2_) lead to an improvement in HER activity, and an increase in the number of active sites. The electron transfer also increased after supporting Pd−MoS_2_ on multiwalled carbon nanotubes (MWNT). These studies demonstrate an excellent HER activity for Pd−MoS_2_/MWNT compared to that observed for free‐standing Pd−MoS_2_ and MoS_2_/MWNT, as well as notable stability in acid media after 500 potential cycles.^[18]^

One of the major drawbacks of carbon‐supported catalysts has been their low durability as defect groups can be introduced by applying electrochemical potentials under strongly acidic conditions.^[19]^ Highly graphitized carbon materials such as carbon nanotubes have been put forward due to their unique corrosion resistance. However, the chemical inertness of the basal plane of a MWNT results in poor dispersion and easy agglomeration of metallic nanoparticles during potential cycling, leading to a rapid decrease of the electrochemically active surface area that detrimentally affects the catalyst activity and durability. Recently, graphitic carbon nitride (g‐C_3_N_4_) has been proposed as a promising alternative due to the presence of a large number of active binding sites (triazine units) for the dispersion of metal nanoparticles, signifying an important step forward in the improvement of the activity and durability of palladium nanoparticles (PdNP) supported on carbon nitride graphene.[^22^, ^23^, ^24^, ^25^, ^26^, ^27^, ^28^, ^29^]

Recently, we have developed an alternative strategy for controlling the activity and durability of electrocatalysts by nanoscale confinement of active binding sites to restrict metal particle migration and coalescence.^[30]^ For example, graphitised carbon nanofibers (GNF) have been effectively utilised as electrocatalytic nanoreactors with their internal step‐edges, consisting of rolled‐up graphitic sheets, acting as effective anchoring points for electrocatalytic nanoparticles and stabilising them during electrochemical cycling.[^30^, ^31^, ^32^, ^33^] The electrocatalytic stability of PtNP supported at the internal step‐edges of GNF was shown to remain at 80 % even after 50000 cycles of the oxygen reduction reaction, significantly outperforming other electrocatalysts, including a commercial Pt/C material studied under the same experimental conditions (0.1 m HClO_4_, 10 mV s^−1^, RT) at the time of the publication.^[33]^

In this study, we report the electrochemical performance of Pd nanoparticles at the graphitic step‐edges of PdNP@GNF electrocatalytic nanoreactors in the HER. The novel catalyst outperforms Pd/C and exhibits a remarkable self‐improving behaviour, becoming more effective with increasing numbers of cycles, eventually surpassing the performance of Pt electrocatalyst analogues. In contrast to previous studies, we demonstrate here that this behaviour is directly linked to interactions between the palladium nanoparticles and the graphitic step‐edges within the GNF, where a reaction between the metal and the carbon is initiated during HER cycling, triggering to hardwire PdNP into the graphitic lattice of the GNF. The resultant change in structure is observed to have a significant impact on both the catalytic activity and durability.

## Results and Discussion

### Preparation and characterisation of PdNP@GNF hybrid electrocatalyst

**Figure 1:**
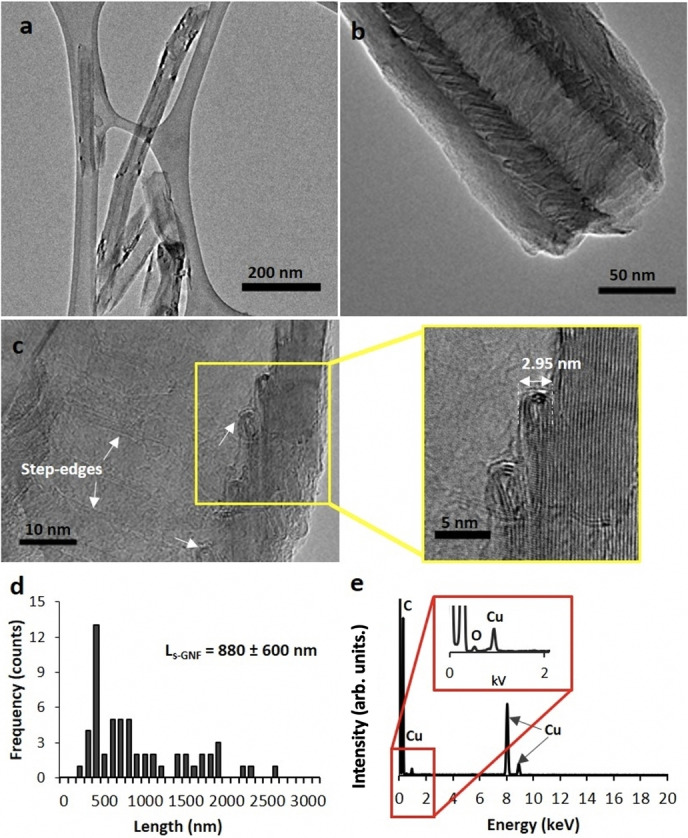
Structural characterisation of shortened GNF. (a–c) HRTEM images of GNF with a high‐magnification view in (c) showing the closed loops of the graphitic step‐edge with a height of approximately 3 nm. (d) GNF length distribution measured by HRTEM (more than 80 measurements) and (e) EDX of GNF after ball milling exhibiting a C/O atomic ratio [%] of 99.5 : 0.5 (Cu peaks are due to the copper TEM support grid).

**Figure 2:**
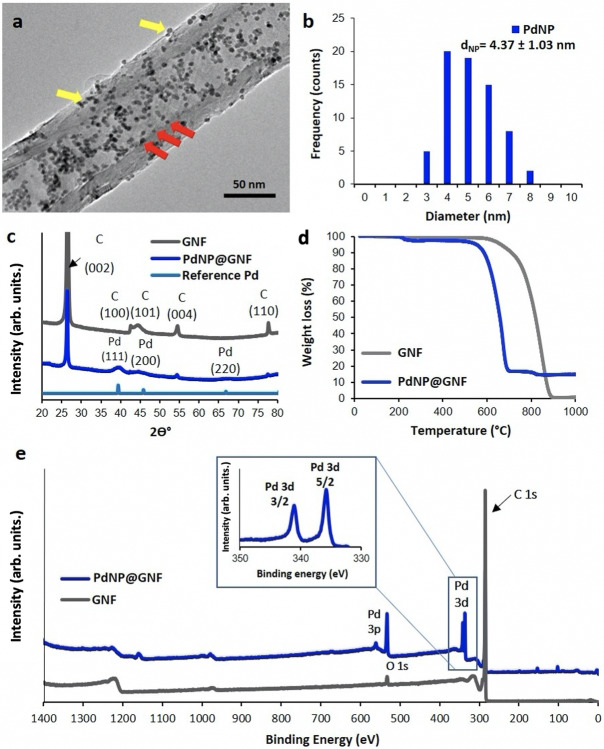
Design and characterisation of PdNP@GNF hybrid electrocatalyst. (a) HRTEM image of PdNP@GNF demonstrating most of the PdNP being encapsulated in the GNF cavities (red arrows), with a minority of PdNP adsorbed on the GNF exteriors (yellow arrows). (b) Size distribution of the PdNP inside and outside the GNF (obtained by measuring more than 80 PdNP in the HRTEM images). (c) Powder XRD patterns of PdNP@GNF, Pd (metal reference 35) and empty GNF. (d) TGA measurements in air of PdNP@GNF (blue) and GNF (green) with 14.1±0.05 % and approximately 0.5 % residual material by weight, respectively. (e) Wide‐scan XPS spectra of GNF and PdNP@GNF with high‐resolution XPS spectra of PdNP@GNF in the Pd 3d regions. Charge correction for the C 1 s spectrum was set at the binding energy of 284.45 eV for each sample.

In order to reduce the length dependence on the mass transport resistance in the HER (Figure 1), GNF were shortened by mechanical ball milling prior to the growth of PdNP, which in turn, also allowed improved film formation on the electrode. Statistical analysis performed on high‐resolution transmission electron microscopy (HRTEM) images confirmed a significant reduction of the mean GNF length from approximately 0.5–60 μm to 880±600 nm by ball milling (Figure 1), while maintaining the integrity of the folded step‐edges of the graphitic planes in their cavities. The PdNP@GNF hybrid was prepared by immersing GNF into a chloroform solution of Pd_2_dba_3_ tris(dibenzylideneacetone)dipalladium(0), which triggered a spontaneous growth of PdNP mainly at the graphitic step‐edges, inside the GNF (see the Experimental Section).^[34]^ The resultant material was imaged by HRTEM confirming the formation of well‐distributed PdNP with an average diameter of 4.37±1.03 nm, with more than 80 % of the PdNP located in the interiors of the GNF (Figure 2a).

HRTEM also confirmed the metallic nature of the PdNP in PdNP@GNF with the spacing of the lattice fringes (0.22 nm) corresponding to the (111) planes of the face‐centred cubic lattice of Pd metal (Figure S1b), in agreement with the diffraction peak centred at 39.5° (2*θ*) in the powder X‐ray diffraction (XRD) measurements (Figure 2c). Comparison of the powder XRD for PdNP@GNF with GNF and a Pd reference further confirm the presence of metallic Pd in the hybrid PdNP@GNF material (Figure 2c).^[35]^ The small size of the PdNP results in the broadening of the XRD peaks as expected with an average size of 3.94±0.51 nm using the Debye‐Scherrer equation, which is in agreement with the value obtained from HRTEM measurements.

The Pd loading in the hybrid material PdNP@GNF was quantified using thermogravimetric analysis (TGA) and shown to be 14.10±0.05 wt%, by measuring the residual weight at 1000 °C in air (Figure 2d and Table S1). TGA studies of PdNP@GNF showed that there was a small weight loss (≈2 %) at around 200 °C, which could be due to the presence of a small amount of residual dibenzylideneacetone (dba) ligand left adsorbed in the sample (see the Experimental Section). A significant decrease in the oxidation temperature of the GNF, from around 680 to 550 °C, was also observed due to the presence of Pd (Figure 2d).

To further confirm the chemical nature of the adsorbed PdNP and evaluate the surface composition of PdNP@GNF, X‐ray photoelectron spectroscopy (XPS) measurements of the hybrid material were carried out by mounting on a Si substrate and compared with empty GNF, as received and shortened (Figure 2e and Figure S2). A very narrow asymmetric C 1 s peak at 284.5 eV was observed with minimal C−O bonding for shortened empty GNF, which was negligible (Table S2) and in agreement with the C/O atomic ratio [%] observed in energy dispersive X‐ray (EDX) spectroscopy (Figure 1d).^[36]^ For the PdNP@GNF sample an oxygen bonded C 1 s peak appears at approximately 284.4 eV, and another one at around 289 eV, which is likely to be a shake‐up feature.^[36]^ The Pd 3d peak at 335.8 eV corresponds to metallic palladium.^[36]^ The O 1 s peak overlaps with the Pd 3p peak at 532 eV, from which the oxygen content was quantified by subtracting the overlapping Pd 3p peak (Table S2).

## Electrocatalysis of the HER in PdNP@GNF nanoreactors

**Figure 3:**
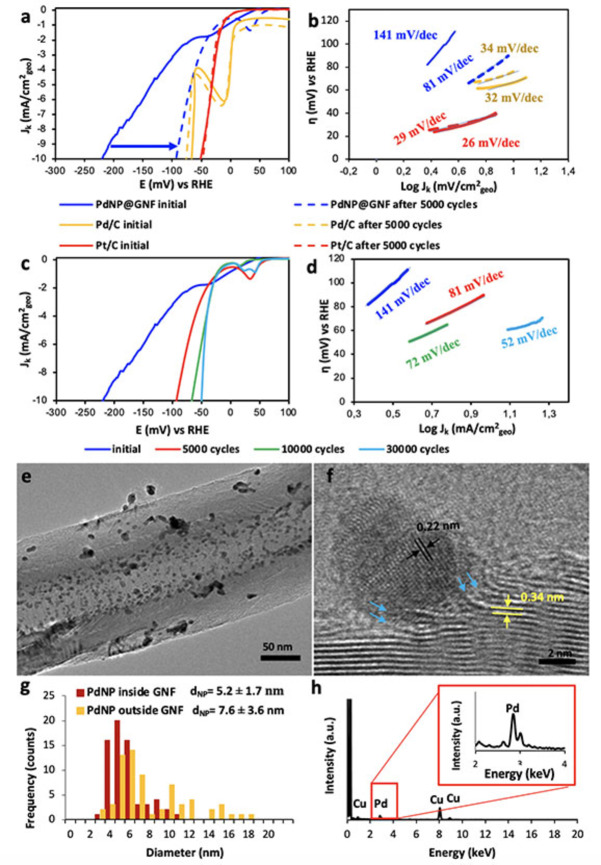
Electrochemical characterisation of PdNP@GNF before and after stability tests in the HER using a carbon rod counter electrode. (a) Comparison of the ohmic drop corrected HER polarisation curves and (b) Tafel plots for the PdNP@GNF electrocatalyst with the commercial benchmarks (Pd/C and Pt/C) before (solid lines) and after (dashed lines) 5000 potential cycles of the HER. The small ripples observed in the polarization curves are due to the accumulation of hydrogen bubbles over time on the surface of the electrode. (c) HER polarization curves and (d) Tafel plots for the PdNP@GNF electrocatalyst material before and after 5000, 10000 and 30000 potential cycles. (e,f) HRTEM images of PdNP@GNF after 30000 HER potential cycles. (f) PdNP bonded to the opened step‐edges (highlighted with blue arrows) and observed *d*‐spacing values between the crystallographic planes for the PdNP (black arrows) (0.22 nm) and graphene layers (yellow arrows) of the GNF (0.34 nm), corresponding to Pd (111) and C (002) planes, respectively. (g) Particle size distributions of PdNP located at the step‐edges (PdNP@GNF) and on the outer surface of the GNF (PdNP/GNF) (measured for more than 80 PdNP per sample), and (h) EDX analysis of PdNP@GNF (Cu peaks are due to the material of TEM sample holder).

Prior to HER measurements, the PdNP@GNF electrode was prepared via dispersion of a PdNP@GNF suspension in hexane using sonication, followed by deposition onto a glassy carbon electrode (GCE) by drop casting (see the Experimental Section). A three‐electrode cell was used for the electrochemical measurements where PdNP@GNF, a carbon rod and a reversible hydrogen electrode (RHE) were used as working, counter and reference electrode, respectively. Commercially available Pd/C (20 wt%) and Pt/C (20 wt%) were selected here as benchmark catalysts towards the HER in which either the PdNP (4.48±0.7 nm) or PtNP (3.07±0.6 nm) are adsorbed on the surface of amorphous carbon or carbon black, respectively, as confirmed by HRTEM images (Figure S3). Both commercial catalysts showed very similar or slightly smaller average particle sizes (in the case of Pt/C), compared to the PdNP in the PdNP@GNF material (Table S1). The electrochemical activity of each catalyst was investigated in HER using linear sweep voltammetry (LSV) measurements between 0.2 and −0.9 V vs. RHE at a sweep rate of 10 mV s^−1^ in a hydrogen‐saturated 0.1 m perchloric acid solution at room temperature (see the Experimental Section). Polarization curves were obtained by plotting the current density (*j*) versus the potential, where the current density was normalised by dividing the current (*i*) by the geometric surface area of the glassy carbon electrode and the potential was corrected by the ohmic resistance in the solution for each catalyst (Figure 3).

Compared to other materials Pt/C displayed the smallest onset potential, very close to 0 mV, in agreement with previous reports.[^22^, ^24^, ^25^, ^26^, ^27^, ^28^, ^29^, ^30^, ^31^, ^32^, ^33^, ^34^, ^35^, ^36^, ^37^] In contrast, PdNP@GNF and commercial Pd/C exhibited more negative onset potentials with similar characteristic polarization curves where two distinct potential ranges can be observed: the first between 30 and −60 mV due to electrochemical hydrogen adsorption and desorption, and the second below −60 mV due to hydrogen evolution (Figure 3a), consistent with the literature.[^22^, ^23^, ^24^, ^25^, ^26^, ^27^, ^28^, ^29^, ^30^, ^31^, ^32^, ^33^, ^34^, ^35^, ^36^, ^37^] The nature of the carbon support appears to have a significant impact on the electrocatalytic performance of the palladium, as PdNP@GNF displays a slightly less negative onset potential of about −56 mV compared to Pd/C (−62 mV), suggesting that the fast electron transport expected for such graphitized nanocarbon structure may be beneficial for electrocatalytic performance. Furthermore, applying the potential required to achieve a current density of −10 mA cm^−2^, often used as a benchmark to compare the electrocatalytic activity of different materials,[^38^, ^39^, ^40^, ^41^] resulted in the following trend Pt/C (47 mV)<Pd/C (76 mV)<PdNP@GNF (221 mV) for our electrocatalysts in their initial states (Figure 3a, Figures S4 and S5, Table S3).

When Pt is used as the electrocatalyst, the Tafel slopes for the Volmer, Heyrovsky, or Tafel steps in acidic electrolytes take values close to 120, 40, or 30 mV dec^−1^ for the rate‐determining step.[^42^, ^43^, ^44^, ^45^, ^46^, ^47^, ^48^] In our work, Pt/C exhibited the best catalytic activity with a Tafel slope of nearly 29 mV dec^−1^, for Pd/C the Tafel slope was only slightly higher (34 mV dec^−1^), whereas for PdNP@GNF the increase was much more pronounced (≈141 mV dec^−1^) (Figure 3). While in Pt/C and Pd/C the Volmer‐Tafel mechanism is the dominant process during the HER showing extremely rapid reaction kinetics (as the recombination of *H*_ads_ is the rate‐determining step), in PdNP@GNF the Heyrowsky step is the rate‐determining step at low overpotential regions (<50 mV) as the Volmer‐Heyrowsky mechanism is dominant. Apart from Tafel slope values, Table S3 collects a summary of the most important electrochemical parameters, including the exchange current density,[^49^, ^50^, ^51^] enabling a complete performance evaluation of the electrocatalyst activity in the HER.

An ideal catalyst is expected to have a low onset potential near to the standard hydrogen reduction potential (0 V_RHE_) combined with a small Tafel slope, but a large exchange current density value.^[11]^ However, some catalysts can exhibit simultaneously a small Tafel slope with a small exchange current density or a high Tafel slope with a high exchange current density. From the Tafel plots, we obtained the highest exchange current density for Pt/C with a value of 0.983 mA cm^−2^_geo_ that is in agreement with the values reported in the literature.[^13^, ^52^, ^53^] The current density for Pt/C was nearly 3 times higher than that observed for PdNP@GNF and nearly 17 times larger than that of Pd/C (Table S3). Despite exhibiting slow HER kinetics, PdNP@GNF has a small onset potential and relatively high current density values compared to Pd/C that can be attributed to the highly conductive nature of the graphitic GNF. Thus, PdNP confined in GNF appear to have an electrochemical behaviour closer to that observed for Pt than Pd on amorphous carbon in conventional electrocatalysts.

### Stability of PdNP@GNF in the HER over 30000 cycles

Long‐term stability is a key factor in determining the practical effectiveness of any electrocatalyst. Here, the stability of PdNP@GNF after 5000 potential cycles was initially probed by continuously applying linear potential sweeps between 0.4 and −0.2 V vs. RHE at 50 mV s^−1^ scan rate and compared with Pd/C and Pt/C benchmark electrocatalysts. Surprisingly, after 5000 HER cycles PdNP@GNF exhibited a remarkable catalytic activity improvement with a reduction of the onset potential and a Tafel slope value of more than 3 times that is in stark contrast to the negligible change in the onset potential and Tafel slopes values observed for Pd/C and Pt/C after potential cycling experiments (Figure 3). Moreover, the largest exchange current density was obtained for PdNP@GNF having increased from 0.276 to 0.386 mA cm^−2^_geo_ during the durability test. In contrast, the exchange current density of Pt/C decreased from 0.983 to 0.880 mA cm^−2^_geo_, whilst Pd/C was slightly improved from 0.058 to 0.069 mA cm^−2^_geo_, a value still significantly lower than those obtained for either PdNP@GNF or Pt/C. Moreover, the change of potential value required to obtain a current density of −10 mA cm^−2^ was consistent with other parameters following the trend PdNP@GNF|128 mV|>Pt/C|19 mV|>Pd/C|9 mV|, which represents a remarkable improvement in the activity of the catalytic centres in PdNP@GNF from the initial state (Table S3). These results not only demonstrate the excellent stability of the PdNP@GNF, but also reveal the unexpected ability of this material to improve its electrocatalytic activity during the HER cycles, in contrast with conventional Pd/C and Pt/C catalyst whose performances either did not change or decreased over time.

To further understand the observed electrocatalytic performance enhancement in the HER, the structure of PdNP@GNF before and after the cycling potential was methodically analysed by HRTEM at the single‐particle level and compared with the commercial electrocatalyst (Pd/C and Pt/C) after 5000 cycles (Figures S3 and S5). Analysis revealed that PdNP adsorbed on the atomically flat external surface of the GNF appear to migrate and grow during potential cycling, in contrast PdNP anchored to the corrugated interior of the GNF remain in the same location with the graphitic step‐edges appearing to prevent nanoparticle migration (Figure 3e). Statistical analysis of the particle size distribution (obtained from HRTEM) confirmed that PdNP anchored at the step‐edges (4.97±1.5 nm) do not measurably change in size after 5000 cycles of the HER; however, PdNP adsorbed on the smooth external surface of the GNF undergo a significant increase in size (7.94±5.1 nm) under the same conditions (Figure S4). A similarly significant nanoparticle growth was observed in Pt/C (6.47±5.2 nm) and Pd/C (8.63±5.3 nm) after 5000 cycles, which correlates with the observed decrease in their electrochemical performance (Figure S5) and further emphasises the importance of the graphitic step‐edges for stabilisation of catalytic metal centres.

**Figure 4:**
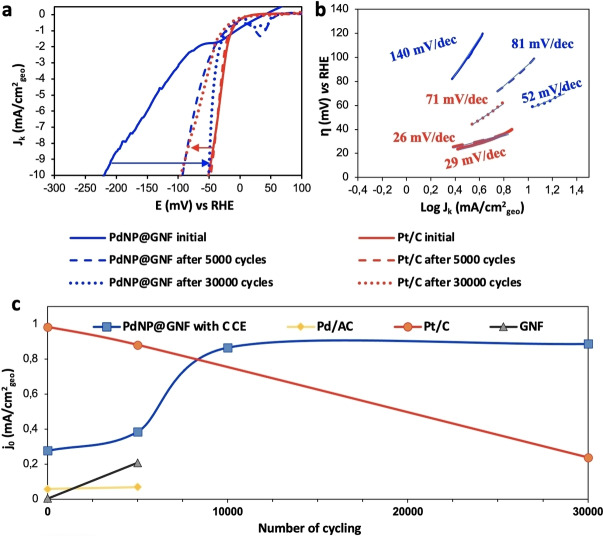
Benchmarking the electrocatalytic performance of PdNP@GNF after stability tests. (a) HER polarization curves and (b) Tafel plots for Pt/C and PdNP@GNF electrocatalyst materials before and after 5000 and 30000 potential cycles using a carbon counter electrode. (c) Comparison of the change on the exchange current densities values of PdNP@GNF, GNF, Pt/C and Pd/C with potential cycling highlighting an increasing trend of the PdNP@GNF HER activity.

Encouraged by the initial HER cycling tests, the electrochemical performance of PdNP@GNF was tested over a larger number of cycles, up to 30000 cycles, and compared with that of the Pt/C electrocatalyst benchmark after 5000 and 30000 cycles under the same conditions (Figure 3c,d and Figure 4). It is worth noting that accelerated durability tests were carried out using a carbon counter electrode, which rule out Pt dissolution‐redeposition effects[^54^, ^55^, ^56^] on the observed electrochemical performance. It is remarkable that as the number of HER cycles increases, the performance of the PdNP@GNF catalyst continues to improve, overtaking Pt/C after about 15000 cycles (Table 1 and Figure 4c). The onset potentials and the Tafel slopes of PdNP@GNF with cycling reached the smallest values after 30000 cycles (−5 mV and 50 mV dec^−1^, respectively), while the exchange current density reached the largest value (0.887 mA cm^−2^_geo_). As shown in Figure 3c, the performance of PdNP@GNF exhibits a remarkable increasing HER activity with a large change in the overpotential value required to achieve a current density of −10 mA cm^−2^ from 221 to 50 mV after 30000 cycles of more than 171 mV. At higher current densities (>|−10 mA cm^−2^|) HER polarization curves show a diffusion current limitation due to both the insufficient mass transport of the produced H_2_ from the catalyst electrode to the bulk electrolyte and the fact that PdNP catalyst is confined in GNF. The former contribution can be minimized under rotation (Figure S6), while the former is restricted to the selected GNF length. Impedance measurements shows that the increase on activity is accompanied with a large decrease in charge transfer resistance (Figure S7). PdNP@GNF also exhibited a similar increase on the HER activity using H_2_SO_4_ instead of HClO_4_ as the electrolyte and under higher concentrations (Figure S8).

**Table 1:** Changes in the onset potential, overpotential, Tafel slopes and exchange current density values in the HER measurements for PdNP@GNF with the number of potential cycles using a carbon counter electrode.

Cycling number	Onset potential [mV]	Overpotential at −10 mA cm^−2^ [mV]	Tafel slope [mV dec^−1^]	*j*_0_ [mA cm^−2^_geo_]
initial	−56	221	140.8	0.276
5000	−17	93	80.9	0.386
10000	−8	87	72.5	0.866
30000	−5	50	51.7	0.887

The dynamics observed for Pt/C is opposite to that of PdNP@GNF as the performance of the former significantly decreased with increasing numbers of cycles. Although the Tafel slope does not change for Pt/C, suggesting that the reaction mechanism remains unchanged, the initial onset potential (0 mV) shifted to −17 mV and the overpotential required to achieve a current density of −10 mA cm^−2^ significantly increased from 47 mV (initial value) to 96 mV after 30000 cycles (Figure 4 and Table S3). Furthermore, a sharp decrease was observed in the exchange current density for Pt/C from an initial value of 0.983 to 0.239 mA cm^−2^_geo_ after the 30000 cycles (Table S3).

The potential cycling experiments up to 30000 cycles clearly reveal the remarkable ability of the PdNP@GNF electrocatalyst to improve its performance during cycling by a factor of three (in terms of *j*_0_ (exchange current density)), which differs drastically to Pt/C and other traditional electrocatalysts that degrade with usage. Interestingly, the significant increase observed for the exchange current density of PdNP@GNF after 5000 cycles matches with the one observed for empty GNF. It is also worth noting that the overpotential value required to achieve a current density of −10 mA cm^−2^ after 30000 potential cycles for the PdNP@GNF (50 mV) is much lower than the one observed for the Pt/C (96 mV). The improvement of HER performance during cycling is also accompanied with an increase of the electrochemical surface area (ECSA), as well as of the mass activity and the specific activity, and a large decrease in resistance that is observed for our material in contrast to the benchmarks (Tables S4–S6 and Figures S9–S12). Surprisingly, after several thousand cycles the electrocatalytic characteristics of confined palladium (PdNP@GNF) in the HER surpass those of platinum in Pt/C, with PdNP@GNF after 30000 cycles exhibiting a better performance than any Pd electrocatalyst reported previously (Table S7).

Both PdNP@GNF and Pt/C were analysed by HRTEM after 30000 cycles. As shown in Figure 3e,f, the PdNP at the step‐edges of GNF are very stable and do not change size significantly (*d*_NP_=5.2±1.7 nm), even after 30000 cycles. In contrast, most of the PdNP adsorbed on the GNF external surfaces appear to agglomerate (*d*_NP_=7.6±3.6 nm) or desorb from the surface during cycling (Figure 3g). In stark contrast, HRTEM images of Pt/C after 30000 cycles clearly show extensive growth of the adsorbed PtNP (Figure S13) that can be attributed to being a result of degradation of the carbon support during the potential cycling (Figure S14). PdNP@GNF was carefully analysed after 30000 HER potential cycles via EDX using scanning transmission electron microscopy (STEM) (Figure S15) clearly showing the location of PdNP. A close inspection of the PdNP adsorbed on the GNF interiors shows that now the step‐edges appear to be unfolded and hardwired to the PdNP (Figure 3f). All these combined observations suggest that the increasing activity with cycling observed for PdNP@GNF may be assigned to the intrinsic electrochemical transformations that seems to be occurring at the folded step‐edges of the GNF triggered by potential cycling.

## The role of the graphitic step‐edges during HER potential cycling

**Figure 5:**
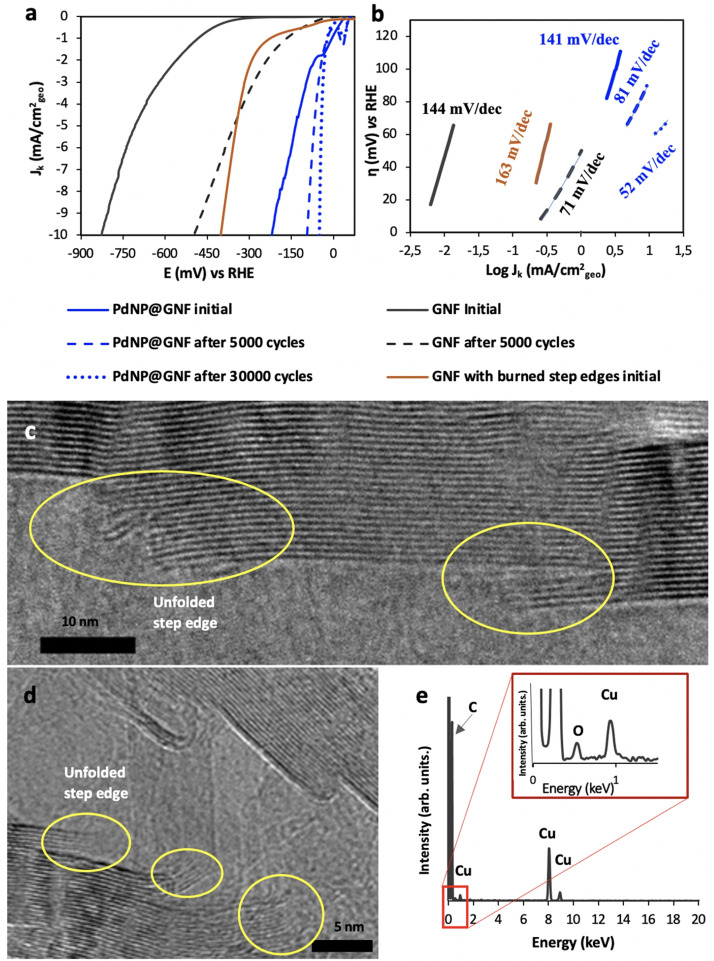
Electrochemical and structural characterisation of the graphitic step‐edges in GNF after HER potential cycling. (a) HER polarization curves and (b) Tafel plots for empty GNF and the PdNP@GNF electrocatalyst before and after stability tests and annealed GNF with open step‐edges using carbon as the counter electrode. (c,d) HRTEM images of empty GNF after 5000 cycles of the HER stability test. In (c,d) the opened step‐edges are circled in yellow. (e) EDX of GNF after ball milling, inset region in the enlarged red rectangle shows the oxygen peak with an atomic ratio [%] of C/O as 99.2 : 0.8.

In order to ascertain the contribution of the GNF support in the electrochemical performance of PdNP@GNF, we investigated the HER activity of empty GNF before and after cycling. The initial HER polarization curves of GNF indicated an onset potential of −320 mV and a potential of 826 mV required to achieve a current density of −10 mA cm^−2^ (Figure 5 and Table S3). A Tafel slope of 143.5 mV dec^−1^ was observed, corresponding to the Volmer‐Heyrowsky mechanism where the Volmer step is the rate‐determining step. Furthermore, a small exchange current density for GNF before cycling is observed (0.004 mA cm^−2^_geo_), which is nearly 60 times lower than that of the initial PdNP@GNF material and 10 times lower than Pd/C (Table S3). The Tafel slopes of GNF before and after 5000 cycles were 143.5 and 71.1 mV dec^−1^, respectively, and a significant increase of the exchange current density is clearly observed (0.208 mA cm^−2^_geo_), which was 44 times higher than the initial value (Figure 5).

It has been hypothesised that the significant improvement in the HER activity of GNF with cycling can only be due to the presence of catalytically active sites containing edge carbon atoms and residual oxygen functional groups promoting electron transfer.[^47^, ^48^, ^57^, ^58^, ^59^, ^60^] To unravel the atomistic mechanism underpinning this process, GNF were carefully investigated by HRTEM after 5000 cycles revealing the unexpected opening of the folded graphitic step‐edges that have undergone transformation during potential cycling (Figure 5c,d). To ascertain the activity enhancement observed to the opening of the step‐edges, GNF were annealed at 600 °C for 5 h in air to selectively open the step‐edges as HRTEM imaging demonstrates (Figure S16) and tested on the HER exhibiting a significant activity enhancement with respect to GNF comparable to that observed for GNF after 5000 cycles (Figure 5a).

The edges of the flat graphene sheets require terminating atoms, which under the HER conditions are likely to be hydrogen atoms. This is in agreement with the hydrogenation process reported through the exposure of graphene to molecular hydrogen gas in the presence of an electric field.^[61]^

Here, we propose that carbon‐carbon bonds in the curved surface of the step‐edge react with hydrogen radicals (H^+^+e^−^) in the Volmer step [Eq. (1)] to form a series of C−H bonds along the zigzag direction of the carbon sheet.^[62]^ This transformation results in the conversion of sp^2^‐hybridised carbons atoms into sp^3^‐hybridised carbons. Further hydrogenation of the step‐edge is promoted by strain relief of the curved surface upon unzipping of the graphitic step edge by C−C bond dissociation along the line of C−H bonds, forming two planar graphitic layers with dangling carbon bonds that in the reaction conditions give rise to the graphitic layers with hydrogen‐terminated edges (Figure S17).

**Figure 6:**
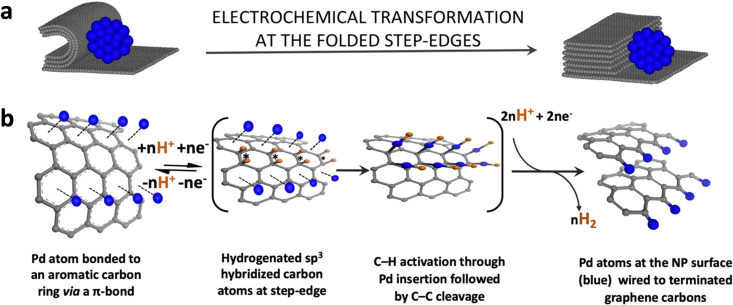
Proposed mechanism of the electrochemical transformation during the HER at the folded step‐edges triggered by potential cycling. (a) Schematic representation of (left) a Pd nanoparticle (blue) attached to a closed step‐edge of a GNF (dark grey) and (right) a Pd nanoparticle wired to an opened step‐edge of a GNF. (b) Reaction scheme of the unzipping process of a closed step‐edge as a result of hydrogenation in the presence of palladium clusters that leads to the formation of covalent σ‐bonds between Pd atoms (blue) at the surface of the NP and edge C‐atoms (grey) of the planar graphitic sheets. Hydrogen atoms are shown in orange. Note that the brackets indicate the intermediate structures and the asterisks indicate positions of the hydrogenated sp^3^ carbons.

All this implies that the observed improvement in the activity of the PdNP@GNF during cycling experiments must result in large part from changes in the GNF carbon support. When PdNP are adsorbed at the step‐edges, they not only provide an active binding site for hydrogen ions to produce molecular hydrogen but may also act as a catalyst to lower the energy barrier for the hydrogenation and rupturing processes at the step‐edge. Knowing the high affinity of palladium for carbon, PdNP can bind to a step‐edge either via strong hybridisation between the Pd d‐orbitals and the carbon π orbitals forming a π‐bond,^[63]^ or direct bonding with the dangling carbon atoms of the open step‐edge via Pd−C σ‐bonds.[^64^, ^65^, ^66^, ^67^] It is proposed that initially the majority of the PdNP are connected to the step‐edges via π‐bonds but over time, as hydrogenation progresses and more C−H bonds are created, stronger σ‐bonds (C−Pd−H) are formed, which is in agreement with HRTEM observations of changes in the PdNP@GNF material after the HER cycling (Figure 6). This leads to an effective hardwiring of palladium atoms into the carbon lattice of the GNF, resulting in improved charge mobility from the macroscopic electrode, through the GNF to the PdNP, and thus, HER activity increasing steadily over the number of cycles. This is also in agreement with the current increase observed for PdNP@GNF when cycling in the hydrogen desorption region (0–0.2 V) (Figure S18).

## Stability studies at constant potential: chronoamperometry experiments

**Figure 7:**
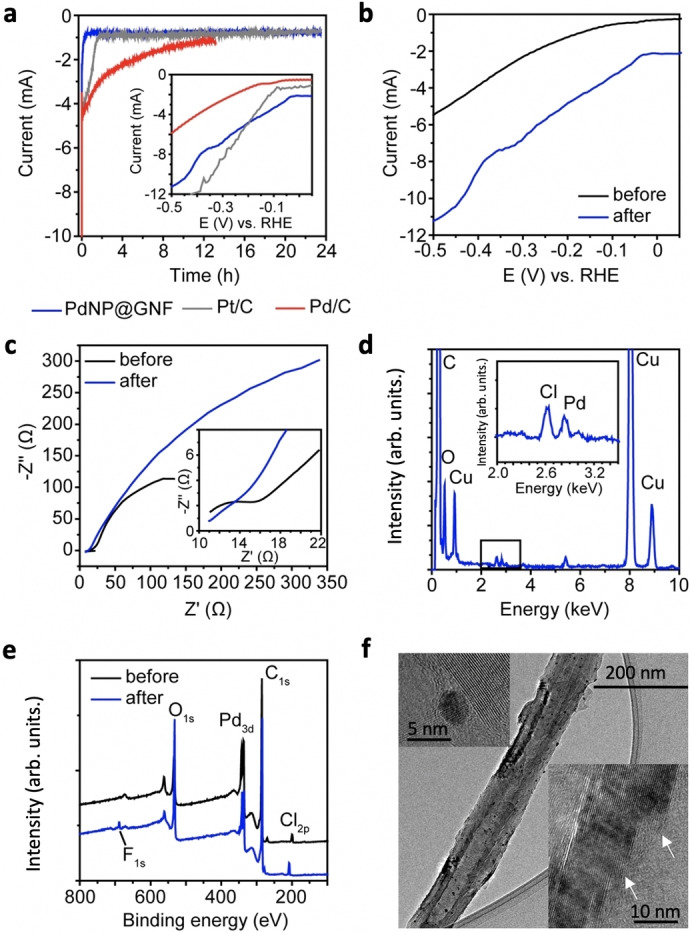
Stability studies at constant potential. (a) 24 h chronoamperometry (CA) experiments for PdNP@GNF electrocatalyst material and the benchmarks (Pt/C and Pd/C) applying a constant voltage of −0.2 V vs. RHE. The inset figure shows the linear sweep voltammograms of each material after CA. (b) HER polarization curves of PdNP@GNF before and after the CA experiment, showing a remarkable HER performance even after 24 h, similar to that observed for Pt/C. (c) Impedance measurements of PdNP@GNF before and after CA. (d) EDX analysis of the sample after CA confirming the presence of palladium with a peak at 2.8 eV (the presence of Cl and Cu is due to the residual HClO_4_ electrolyte and the use of a lacey carbon film coated copper grid for the measurements). (e) XPS survey spectra of PdNP@GNF before and after CA shows the presence of Pd 3d and F 1 s after the measurement due to the Nafion solution used to make the electrode. (f) HRTEM image of a PdNP@GNF nanohybrid. Figure insets show higher magnifications of the sample where opened step edges and confined Pd nanoparticles (with an average NP size of 5.10±1.48 nm) can be seen.

To assess whether the observed trend on the HER electrochemical performance of our PdNP@GNF electrode material applies also for constant potential experiments, we carried out chronoamperometry measurements for 24 h using a carbon rod as the counter electrode for PdNP@GNF and compare with Pt/C and Pd/C benchmarks in the same conditions (Figures S19 and S20). As shown in Figure 7a, our electrocatalyst material shows a remarkable performance for the HER under an applied potential of −0.2 V vs. RHE that is very similar to that observed for Pt/C benchmark and far better than that observed for Pd/C. Complementary electrochemical and structural characterisation measurements before and after the 24 h chronoamperometry experiment of the PdNP@GNF electrode (Figure 7b–f) indicate that the observed increase on the HER activity is concomitant with the opening of the step‐edges and a decrease of the resistance, as shown by TEM and impedance measurements, respectively. XPS and EDX measurements show no changes in the composition or nature of the PdNP in the PdNP@GNF electrode (Figure S21). The small change in the average nanoparticle size of the confined PdNP observed after 24 h chronoamperometry [from 4.37±1.03 nm (initial) to 5.10±1.48 nm] is similar to that observed under potential cycling (5.2±1.7 nm after 30000 cycles). All these combined measurements suggest that the observed behaviour is in agreement with the stability studies carried out by cycling.

## Conclusions

While the performance of most catalysts degrades over time, in this study we present a catalytic nanoreactor PdNP@GNF (palladium nanoparticles supported inside hollow graphitised carbon nanofibers), which exhibits the opposite trend, improving it performance during the reaction. Over 30000 cycles of the hydrogen evolution reaction (HER), the activity of palladium nanoparticles confined within the carbon nanoreactor continually increases, surpassing other palladium catalysts and matching that of platinum. The nanoscale confinement of nanoparticles at the graphitic step‐edges in carbon nanoreactors was found to play a crucial role in the self‐improving electrocatalyst. Detailed high‐resolution transmission electron microscopy imaging reveals important structural changes at the nanoscale in the hybrid electrocatalyst PdNP@GNF taking place under accelerated stability tests and unravels the orchestrated series of reactions occurring at the step‐edges within nanoreactors. During both electrochemical cycling and chronoamperometry (holding at constant potential), carbon bonds unzip to form flat graphene layers that enable highly effective bonding of Pd atoms to the opened step‐edges, allowing more efficient electron transport from the GNF to the metal. This unique, superior durability and catalytic activity of PdNP@GNF, increasing over 30000 HER potential cycles, leads to the most effective Pd‐electrocatalyst for HER reported to date. Detailed, atomic‐level structural analysis and carefully designed control experiments allowed us to assign the unique electrocatalytic behaviour of PdNP@GNF to location specific interactions between the PdNP and the graphitic step‐edges induced by either electrocatalytic potential cycling or chronoamperometry, resulting in hardwiring of the PdNP into the graphitic lattice. We demonstrate that the utilisation of catalytically active nanoparticles inside carbon nanoreactors provides an effective solution to the challenge of enhancing the activity and durability of electrocatalysts. In summary, these materials have the potential to offer a sustainable use of precious metals in hydrogen generation and related electrochemical processes, key targets, if we are to address the global economic and social need for advancement of zero‐emission technologies.

## Experimental Section

GNF were purchased from Pyrograf Products Inc. Nafion® (5 % solution in a mixture of lower aliphatic alcohols and water) and all other reagents and solvents were purchased from Sigma‐Aldrich (UK) and used without further purification. Pt/C (20 wt%) and Pd/C commercial catalysts were supplied by Johnson Matthey and Alfa Aesar, respectively. Ultra‐pure water purified with Millipore Advantage A10 water equipment (resistivity 18.2 MΩ cm at 25 °C) was used in all experiments. HRTEM analysis was performed on a JEOL 2100 Field emission gun microscope with an information limit of 0.12 nm at 200 kV and a JEOL JEM F200 microscope equipped with a cold field‐emission gun (Cold‐FEG) operated at 200 kV with an high‐resolution pole piece. TEM images were acquired using a Gatan OneView camera. EDX was performed with a Centurio Large Angle Silicon Drift Detector (SDD) that collects X‐rays from a detection area of 100 mm^2^. Samples for HRTEM analysis were prepared by dispersing the materials in HPLC grade *iso*‐propanol using ultrasonication, then drop casting the resultant suspension onto a lacey carbon film coated copper grid. Microwave acid digestion of the sample was carried out before inductively coupled plasma optical emission spectroscopy (ICP‐OES) analysis. TGA analysis was performed on a TA Instruments TGA‐SDTQ600 analyser in air up to 1000 °C with a heating rate of 10 °C min^−1^. The powder XRD patterns were obtained using a PANanalytical X'Pert PRO diffractometer equipped with a CuK_α_ radiation source (*λ*=1.5418 Å) operating at 40 kV and 40 mA, with a 0.05252° step size and a 5925.18 s step time. XPS measurements were carried out using a Kratos AXIS ULTRA spectrometer with a mono‐chromated AlK_α_ X‐ray source (1486.6 eV) operated at 10 mA emission current and 12 kV anode potential (120 W). The electrochemical experiments were performed in 0.1 m HClO_4_ using a three‐electrode set‐up and an Autolab potentiostat PGSTAT204 with a GCE, RHE and a carbon rod as working, reference and counter electrode, respectively. For chronoamperometry experiments, a smaller‐volume three‐electrode set‐up cell using Ag/AgCl reference electrode, carbon rod as counter electrode and a glassy carbon working electrode were employed. The current during chronoamperometry and LSV before and after the experiment were measured using a high‐voltage power supply. The GCE was cleaned by polishing with 0.05 μm principal particle size alumina powder solution (Agar Scientific Ltd.).

### Shortening GNF via ball milling

GNF (50 mg) as received, with a length of approximately 15±12 μm,^[68]^ were mechanically ground in an ambient atmosphere using a Retsch MM 400 ball mill instrument (10 Hz for 1.5 h), containing a steel ball with a diameter of 10 mm.

### Preparation of PdNP@GNF

Shortened GNF (15 mg) were dispersed in CHCl_3_ (40 mL) under sonication for 10 min. A solution of Pd_2_dba_3_ ⋅ CHCl_3_ (11.2 mg) in CHCl_3_ (20 mL) was then slowly added to the GNF dispersion in small portions (0.5 mL) whilst being treated with ultrasonic waves. The mixture was further sonicated for 10 min. It was then magnetically stirred at 40 °C for 1 day until the supernatant solution became colourless. The PdNP@GNF was then separated from the reaction mixture by filtration and washed repeatedly with acetone (20 mL) using a 0.2 μm polytetrafluoroethylene membrane filter to remove the free dba and obtain the final PdNP@GNF material as a black powder.

## Supporting Information

Supporting Information is available from Wiley Online Library. Further details of the electrochemical measurements including chronoamperometry studies and experiments done using carbon rod as CE. Additional tables and figures showing TGA measurements, HRTEM images and particle size distributions for PdNP@GNF, Pd/C, and Pt/C, XPS data for GNF and PdNP@GNF and further electrochemical measurements, and analysis of the performances of PdNP@GNF, the standards (Pd/C, Pt/C) and GNF before and after potential cycling, including STEM‐EDX elemental mapping of PdNP@GNF after 30000 potential cycles.

## Conflict of interest

The authors declare no conflict of interest.

## Supporting information

As a service to our authors and readers, this journal provides supporting information supplied by the authors. Such materials are peer reviewed and may be re‐organized for online delivery, but are not copy‐edited or typeset. Technical support issues arising from supporting information (other than missing files) should be addressed to the authors.

Supporting Information

## References

[1] M. Caban-Acevedo , M. L. Stone , J. Schmidt , J. G. Thomas , Q. Ding , H. C. Chang , M. L. Tsai , J.-H. He , S. Jin , Nat. Mater. 2015, 14, 1245–1251. 26366849 10.1038/nmat4410

[2] S. Cobo , J. Heidkamp , P. A. Jacques , J. Fize , V. Fourmond , L. Guetaz , B. Jousselme , V. Ivanova , H. Dau , S. Palacin , M. Fontecave , V. Artero , Nat. Mater. 2012, 11, 802–807. 22863815 10.1038/nmat3385

[3] M. Dresselhaus , I. Thomas , Nature 2001, 414, 332–337. 11713539 10.1038/35104599

[4] R. M. Navarro , M. A. Pena , J. L. G. Fierro , Chem. Rev. 2007, 107, 3952–3991. 17715983 10.1021/cr0501994

[5] Y. Jiao , Y. Zheng , M. Jaroniec , S. Z. Qiao , Chem. Soc. Rev. 2015, 44, 2060–2086. 25672249 10.1039/c4cs00470a

[6] J. D. Benck , T. R. Hellstern , J. Kibsgaard , P. Chakthranont , T. F. Jaramillo , ACS Catal. 2014, 4, 3957–3971.

[7] H. L. Fei , J. C. Dong , M. J. Arellano-Jimenez , G. L. Ye , N. D. Kim , E. L. G. Samuel , Z. W. Peng , Z. Zhu , F. Qin , J. M. Bao , M. J. Yacaman , P. M. Ajayan , D. L. Chen , J. M. Tour , Nat. Commun. 2015, 6, 8668. 26487368 10.1038/ncomms9668 PMC4639894

[8] H. A. Gasteiger , S. S. Kocha , B. Sompalli , F. T. Wagner , Appl. Catal. B 2005, 56, 9–35.

[9] H. A. Gasteiger , N. M. Marković , Science 2009, 324, 48–49. 19342578 10.1126/science.1172083

[10] S.-K. Park , D. Y. Chung , D. Ko , Y.-E. Sung , Y. Piao , J. Mater. Chem. A 2016, 4, 12720–12725.

[11] S. Lu , Z. Zhuang , Sci. China Mater. 2016, 59, 217–238.

[12] J. Durst , C. Simon , F. Hasche , H. A. Gasteiger , J. Electrochem. Soc. 2015, 162, F190-F203.

[13] Y. Cheng , S. Lu , F. Liao , L. Liu , Y. Li , M. Shao , Adv. Funct. Mater. 2017, 27, 1700359, 1–6.

[14] N. C. Cheng , N. B. Banis , J. Liu , A. Riese , X. Li , R. Y. Li , S. Y. Ye , S. N. Knights , X. L. Sun , Adv. Mater. 2015, 27, 277–281. 25405600 10.1002/adma.201404314

[15] Y. Xu , M. Kraft , R. Xu , Chem. Soc. Rev. 2016, 45, 3039–3052. 27094875 10.1039/c5cs00729a

[16] D. V. Esposito , S. T. Hunt , A. L. Stottlemyer , K. D. Dobson , B. E. McCandless , R. W. Birkmire , J. G. G. Chen , Angew. Chem. Int. Ed. 2010, 49, 9859–9859; 10.1002/anie.201004718 20886586

[17] S. Liu , X. Mu , H. Duan , C. Chen , H. Zhang , Eur. J. Inorg. Chem. 2017, 535–539.

[18] E. Heydari-Bafrooei , S. Askari , Int. J. Hydrogen Energy 2017, 42, 2961–2969.

[19] L. Zhang , Q. Chang , H. Chen , M. Shao , Nano Energy 2016, 29, 198–219.

[20] J. Li , F. Li , S. X. Guo , J. Zhang , J. Ma , ACS Appl. Mater. Interfaces 2017, 9, 8151–8160. 28198611 10.1021/acsami.7b01241

[21] S. A. Grigoriev , P. Millet , V. N. Fateev , J. Power Sources 2008, 177, 281–285.

[22] T. Bhowmik , M. K. Kundu , S. Barman , ACS Catal. 2016, 6, 1929–1941.

[23] Y. Zhao , F. Zhao , X. P. Wang , C. Y. Xu , Z. P. Zhang , G. Q. Shi , L. T. Qu , Angew. Chem. Int. Ed. 2014, 53, 13934–13939; 10.1002/anie.201409080 25381722

[24] M. Shalom , S. Gimenez , F. Schipper , I. Herraiz-Cardona , J. Bisquert , M. Antonietti , Angew. Chem. Int. Ed. 2014, 53, 3654–3658; 10.1002/anie.201309415 24574144

[25] J. T. Zhang , L. T. Qu , G. Q. Shi , J. Y. Liu , J. F. Chen , L. M. Dai , Angew. Chem. Int. Ed. 2016, 55, 2230–2234; 10.1002/anie.201510495 26709954

[26] Y. Ito , W. T. Cong , T. Fujita , Z. Tang , M. W. Chen , Angew. Chem. Int. Ed. 2015, 54, 2131–2136; 10.1002/anie.201410050 25470132

[27] J. J. Duan , S. Chen , M. Jaroniec , S. Z. Qiao , ACS Nano 2015, 9, 931–940. 25559360 10.1021/nn506701x

[28] Y. Jiao , Y. Zheng , K. Davey , S. Z. Qiao , Nat. Energy 2016, 1, 16130.

[29] D.-W. Wang , D. Su , Energy Environ. Sci. 2014, 7, 576–591.

[30] M. C. Gimenez-Lopez , A. La Torre , M. W. Fay , P. D. Brown , A. N. Khlobystov , Angew. Chem. Int. Ed. 2013, 52, 2051–2054; 10.1002/anie.201207855 23307656

[31] A. La Torre , M. C. Gimenez-Lopez , M. W. Fay , G. A. Rance , W. A. Solomonsz , T. W. Chamberlain , P. D. Brown , A. N. Khlobystov , ACS Nano 2012, 6, 2000–2007. 22356571 10.1021/nn300400z

[32] A. La Torre , M. C. Gimenez-Lopez , M. W. Fay , C. H. Lucas , P. D. Brown , A. N. Khlobystov , Small 2015, 11, 2756–2761. 25689488 10.1002/smll.201402807

[33] M. C. Gimenez-Lopez , A. Kurtoglu , D. A. Walsh , A. N. Khlobystov , Adv. Mater. 2016, 28, 9103–9108. 27571503 10.1002/adma.201602485

[34] M. Aygün , T. W. Chamberlain , M. C. Gimenez-Lopez , A. N. Khlobystov , Adv. Funct. Mater. 2018, 28, 1802869.

[35] L. Xu , X.-C. Wu , J.-J. Zhu , Nanotechnology 2008, 19, 30, 305603. 10.1088/0957-4484/19/30/305603 21828765

[36] G. Beamson , D. Briggs , J. Chem. Educ. 1993, 70, 1, A25.

[37] A. Zalineeva , S. Baranton , C. Coutanceau , G. Jerkiewicz , Langmuir 2015, 31, 1605–1609. 25068587 10.1021/la5025229

[38] J. D. Benck , T. R. Hellstern , J. Kibsgaard , P. Chakthranont , T. F. Jaramillo , ACS Catal. 2014, 4, 3957–3971.

[39] J. Li , G. Zheng , Adv. Sci. 2017, 4, 1600380. 10.1002/advs.201600380 PMC5357991 28331791

[40] Y. J. Tang , Y. Wang , X. L. Wang , S. L. Li , W. Huang , L. Z. Dong , Adv. Energy Mater. 2016, 6, 1600116.

[41] C. G. Morales-Guio , L. A. Stern , X. Hu , Chem. Soc. Rev. 2014, 43, 6555–6569. 24626338 10.1039/c3cs60468c

[42] J. O. M. Bockris , E. C. Potter , J. Electrochem. Soc. 1952, 99, 169.

[43] A. J. Bard , L. R. Faulkner , Electrochemical Methods: Fundamentals and Applications, 2nd *ed*., Wiley, New York, 2001, 580–632.

[44] J. Janata , Angew. Chem. Int. Ed. 2011, 50, 41, 9538–9538.

[45] D. K. Singh , R. N. Jenjeti , S. Sampath , M. Eswaramoorthy , J. Mater. Chem. A 2017, 5, 6025–6031.

[46] Y. Liu , H. Yu , X. Quan , S. Chen , H. Zhao , Y. Zhang , Sci. Rep. 2014, 4, 6843. 25354806 10.1038/srep06843 PMC4213799

[47] B. Conway , B. Tilak , Electrochim. Acta 2002, 47, 3571–3594.

[48] M. Gong , W. Zhou , M. C. Tsai , J. Zhou , M. Guan , M. C. Lin , B. Zhang , Y. Hu , D. Y. Wang , J. Yang , S. J. Pennycook , B. J. Hwang , H. Dai , Nat. Commun. 2014, 5, 4695. 25146255 10.1038/ncomms5695

[49] A. R. Kucernak , C. Zalitis , J. Phys. Chem. C 2016, 120, 10721–10745.

[50] M. R. Gao , J. X. Liang , Y. R. Zheng , Y. F. Xu , J. Jiang , Q. Gao , J. Li , S. H. Yu , Nat. Commun. 2015, 6, 5982. 25585911 10.1038/ncomms6982 PMC4309426

[51] Q. Lu , G. S. Hutchings , W. Yu , Y. Zhou , R. V. Forest , R. Tao , J. Rosen , B. T. Yonemoto , Z. Cao , H. Zheng , J. Q. Xiao , F. Jiao , J. G. Chen , Nat. Commun. 2015, 6, 6567, 1–8. 25910892 10.1038/ncomms7567 PMC4382682

[52] N. M. Marković , B. N. Grgur , P. N. Ross , J. Phys. Chem. B 1997, 101, 5405–5413.

[53] Y. Han , X. Yue , Y. Jin , X. Huang , P. K. Shen , J. Mater. Chem. A 2016, 4, 10, 3673–3677.

[54] L. Ring , B. G. Pollet , M. Chatenet , S. Abbou , K. Küpper , M. Schmidt , M. Huck , A. Gries , M. Steinhart , H. Schäfer , Angew. Chem. Int. Ed. 2019, 58, 17383–17392; 10.1002/anie.201908649 PMC7155044 31539189

[55] M. A. Bird , S. E. Goodwin , D. A. Walsh , ACS Appl. Mater. Interfaces 2020, 12, 18, 20500–20506. 10.1021/acsami.0c03307 32282181

[56] J. Zhu , L. Hu , P. Zhao , L. Y. S. Lee , K.-Y. Wong , Chem. Rev. 2020, 120, 2, 851–918. 10.1021/acs.chemrev.9b00248 31657904

[57] D. Deng , K. S. Novoselov , Q. Fu , N. Zheng , Z. Tian , X. Bao , Nat. Nanotechnol. 2016, 11, 218–230. 26936816 10.1038/nnano.2015.340

[58] Y. Tian , Y. Ye , X. Wang , S. Peng , Z. Wei , X. Zhang , W. Liu , Appl. Catal. A 2017, 529, 127–133.

[59] M. Wang , Z. Wang , X. Gong , Z. Guo , Renewable Sustainable Energy Rev. 2014, 29, 573–588.

[60] S. Pal , M. Sahoo , V. T. Veettil , K. K. Tadi , A. Ghosh , P. Satyam , R. K. Biriju , P. M. Ajayan , S. K. Nayak , T. N. Narayanan , ACS Catal. 2017, 7, 2676–2684.

[61] Z. Ao, S. Li, (August 1st 2011). Hydrogenation of Graphene and Hydrogen Diffusion Behavior on Graphene/Graphane Interface, *Graphene Simulation* **2011**, 53–73, Edited by Jian Ru Gong, IntechOpen, DOI: 10.5772/20676.

[62] J. Berashevich , T. Chakraborty , Phys. Rev. B 2011, 83, 195442.

[63] C. Gong , D. Hinojos , W. Wang , N. Nijem , B. Shan , R. M. Wallace , K. Cho , Y. J. Chabal , ACS Nano 2012, 6, 5381–5387. 22540140 10.1021/nn301241p

[64] G. M. Psofogiannakis , G. E. Froudakis , Chem. Commun. 2011, 47, 7933–794. 10.1039/c1cc11389e 21528146

[65] V. B. Parambhath , R. Nagar , K. Sethupathi , S. Ramaprabhu , J. Phys. Chem. C 2011, 115, 15679–1568.

[66] C. I. Contescu , C. M. Brown , Y. Liu , V. V. Bhat , N. C. Gallego , J. Phys. Chem. C 2009, 113, 5886–5890.

[67] Z. Chen , E. Vorobyeva , S. Mitchell , E. Fako , M. A. Ortuño , N. Lopez , S. M. Collins , P. A. Midgley , S. Richard , G. Vile , J. Perez-Ramirez , Nat. Nanotechnol. 2018, 13, 702–707. 29941887 10.1038/s41565-018-0167-2

[68] M. Aygün , C. T. Stoppiello , M. Lebedeva , E. Smith , M. C. Gimenez-Lopez , A. Khlobystov , T. W. Chamberlain , J. Mater. Chem. A 2017, 5, 21467–21477.

