# Supplementary material for: Palladium Nanoparticles Hardwired in Carbon Nanoreactors Enable Continually Increasing Electrocatalytic Activity During the Hydrogen Evolution Reaction
Source: ChemSusChem. 2021 Sep 15;14(22):4973–84. doi: 10.1002/cssc.202101236 (PMC9292725; doi:10.1002/cssc.202101236)
Supplement: Supplementary file 1 — Supporting Information [file CSSC-14-4973-s001.pdf]

ChemSusChem

Supporting Information

Palladium Nanoparticles Hardwired in Carbon Nanoreactors Enable Continually Increasing Electrocatalytic Activity During the Hydrogen Evolution Reaction

Mehtap Aygün[+], Melanie Guillen-Soler[+], Jose M. Vila-Fungueiriño, Abdullah Kurtoglu, Thomas W. Chamberlain, Andrei N. Khlobystov,* and Maria del Carmen Gimenez-Lopez*© 2021 The Authors. ChemSusChem published by Wiley-VCH GmbH. This is an open access article under the terms of the Creative Commons Attribution License, which permits use, distribution and reproduction in any medium, provided the original work is properly cited.

Supplementary Information

Palladium Nanoparticles Hardwired in Carbon Nanoreactors Enable Continually Increasing Electrocatalytic Activity During the Hydrogen Evolution Reaction

Mehtap Aygün[1,2‡], Melanie Guillen-Soler[1‡], Jose M. Vila-Fungueiriño[1], Abdullah Kurtoglu[3], Thomas W. Chamberlain[4], Andrei N. Khlobystov[5], Maria del Carmen Gimenez-Lopez[1*]*

[1]*Centro Singular de Investigación en Química Biolóxica e Materiais Moleculares (CIQUS), Universidade de Santiago de Compostela, 15782 Santiago de Compostela, Spain.*

[2] *Present address: Faculty of Science, Erzurum Technical University, Erzurum, 25050, Turkey.*

[3]*School of Chemistry, University of Nottingham, University Park, Nottingham, NG7 2RD, UK.*

[4]*Institute of Process Research and Development, School of Chemistry, University of Leeds, Leeds, LS2 9JT, UK.*

[5]*Nanoscale & Microscale Research Centre, University of Nottingham, University Park, Nottingham, NG7 2RD, UK.*

[‡]*These authors contributed equally to this work.*

E-mail: Maria.Gimenez.Lopez@usc.es; Andrei. Khlobystov@nottingham.ac.uk

Electrochemical measurements

Film electrode preparation: The electrocatalyst material (1 mg) was dispersed in hexane (1 mL) using ultrasonication for 15 minutes. The obtained ink was deposited onto the GCE by drop casting, with the resultant film covered by a Nafion solution (0.06%) and then dried under argon flow at room temperature for 30 min. All the electrocatalyst films were prepared using the same procedure with a resultant metal loading of 100 $\mu g/cm^2_{geo}$ for all of them.

Impedance measurements: The cell resistance was measured immediately before HER measurements taking the impedance spectra from 32 to 0.1 kHz and a voltage perturbation of 10 mV. The real part of the resistance at 1 kHz was taken as the cell resistance and was used to obtain the IR-free potential of the working electrode. All of the I-E curves was corrected by the solution resistance using $E = E_{experimental} - (I*R)$.

Tafel slopes: Slopes were calculated from the slope of the linear fitting in the polarization curves using the Tafel equation simplified from the Butler-Volmer equation:

$$\eta_a = \frac{0.059}{(1-\alpha_c)n} log(j_0) - \frac{0.059}{(1-\alpha_c)n} log(j) \quad \text{at 25 °C.}$$

Here η is the overpotential (η = E_0-E, i.e. the difference between the applied potential (E) and the equilibrium potential (E_0) *vs* the RHE, which is 0 for HER in acid), J_0 is the exchange current density at 0 V overpotential. This equation can be simplified to;

$$\eta = a + b\ log(j)$$

It indicates a linear relationship between the overpotential and log (*j*) and thus a slope *b* is generated and described as the Tafel slope when the overpotential is plotted as a function of log (*j*).

Electrochemical active surface area (ECSA) values were obtained with an integration of the peak area of hydrogen adsorption region (Hupd) by subtracting the double-layer charging currents via cyclic voltammetry method.

$$Q_H[C] = \frac{Peak\ area\ [A \cdot V]}{Scan\ rate\ [\frac{V}{s}]}$$

$$ECSA\left[\frac{m^2}{g}\right] = \frac{Q_H[C]}{C\ \left[\frac{\mu C}{cm^2}\right] * L_{metal}\ \left[\frac{mg_{metal}}{cm^2}\right] * A_g\ [cm^2]} * 10^5$$

where, Q_H is the total charge obtained from CV; L_{metal} is the loading of metal catalyst on 1 cm^2 of glassy carbon electrode; C is the charge required to reduce a monolayer of protons on the metal (for Pt 210 μC/cm^2, for Pd 240 μC/cm^2) and A_g is the geometric surface area of the glassy carbon working electrode.

The MA of the catalyst is calculated by the followed equation:

$$MA[\text{A/mg}] = \frac{r_f i_k}{L_{Pd}}$$

The roughness factor (r_f) is calculated from the equation below. The ratio between the real metal surface area A_{real} measured from CV and the geometric area of the electrode A_{geo} obtained from the surface area of the working electrode is equal to 0.196 cm^2 .

$$r_f = \frac{A_{real}}{A_{geo}} = \frac{Q_H(C)}{\frac{240(\frac{\mu C}{cm^2})}{0,196\ (cm^2)}}$$

The specific activity (SA) for the current per unit surface area of catalyst was obtained by the following formula:

$$SA[mA/cm^2] = \frac{MA\left[\frac{A}{mg}\right] x\ 100}{ECSA\ [m^2/g]}$$

Chronoamperometry measurements: A constant potential of -0.2 V vs RHE was applied for PdNP@GNF and Pt/C for 24 hours, while for Pd/C the same potential was applied for 13 hours. A three set-up cell (**Supplementary Figure 19**) with a glassy carbon working electrode (0.5 cm^2), carbon rod counter electrode (**Supplementary Figure 20)** and Ag/AgCl as reference electrode was used. A solution of 0.1M $HClO_4$ was employed as the electrolyte. The solution was degassed for 15 min with argon. A magnetic stirrer was used during the experiment to help to remove bubbles on the surface of the electrode. To prepare the electrode, 3 mg of the material were sonicated in 1 mL of hexane to make an ink, where 90 uL of this ink were deposited on the GC. After drying, Nafion solution (40 mL) was added on the surface. The electrode was left to dry.

The general equation for this conversion at any pH using Ag/AgCl reference electrode is:

$$\mathbf{E_{(RHE)} = E_{Ag/AgCl} + 0.059\ pH + E^o_{Ag/AgCl}}$$

Where $E^o_{Ag/AgCl}$ = 0.1976 V at 25°C and $E_{Ag/AgCl}$ is the working potential

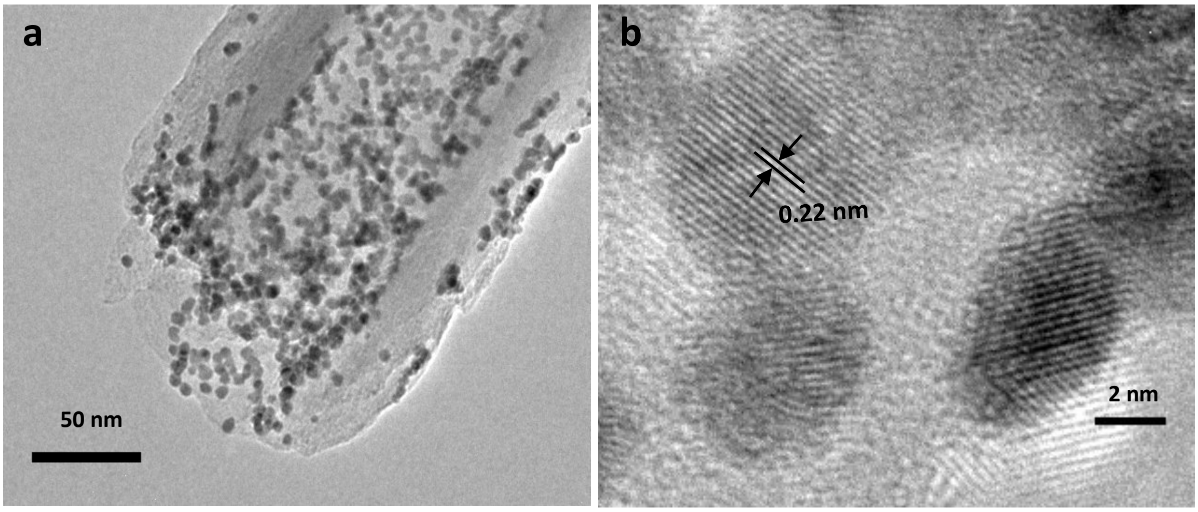

Supplementary Figure 1 | (**a**) HRTEM images of PdNP@GNF in which most of the PdNP are observed to be encapsulated in the GNF cavities. (**b**) The crystal lattice of the PdNP with a distance between adjacent planes of 0.22 nm corresponds to the (111) peak centred at 39.5° (as 2Θ) in the XRD measurements.

Supplementary Table 1| Summary of the average values for nanoparticle size obtained as the mean diameter assuming a spherical shape and metal loading (%) by weight for PdNP@GNF, Pd/C and Pt/C.

Catalyst	NP size (nm)	Metal loading (%wt)
**PdNP@GNF**	4.37 ± 1.03	14*
**Pd/C**	4.48 ± 0.7	20
**Pt/C**	3.07 ± 0.6	20

*Metal loading is determined by TGA. Please note that for PdNP@GNF the small weight loss (~ 2%) observed at ~ 200 °C can be assigned to the loss of residual dba as shown in the below TGA of pure dba (scan rate: 10°C/min, air).

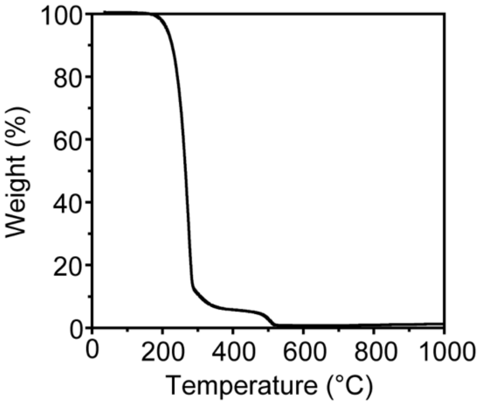

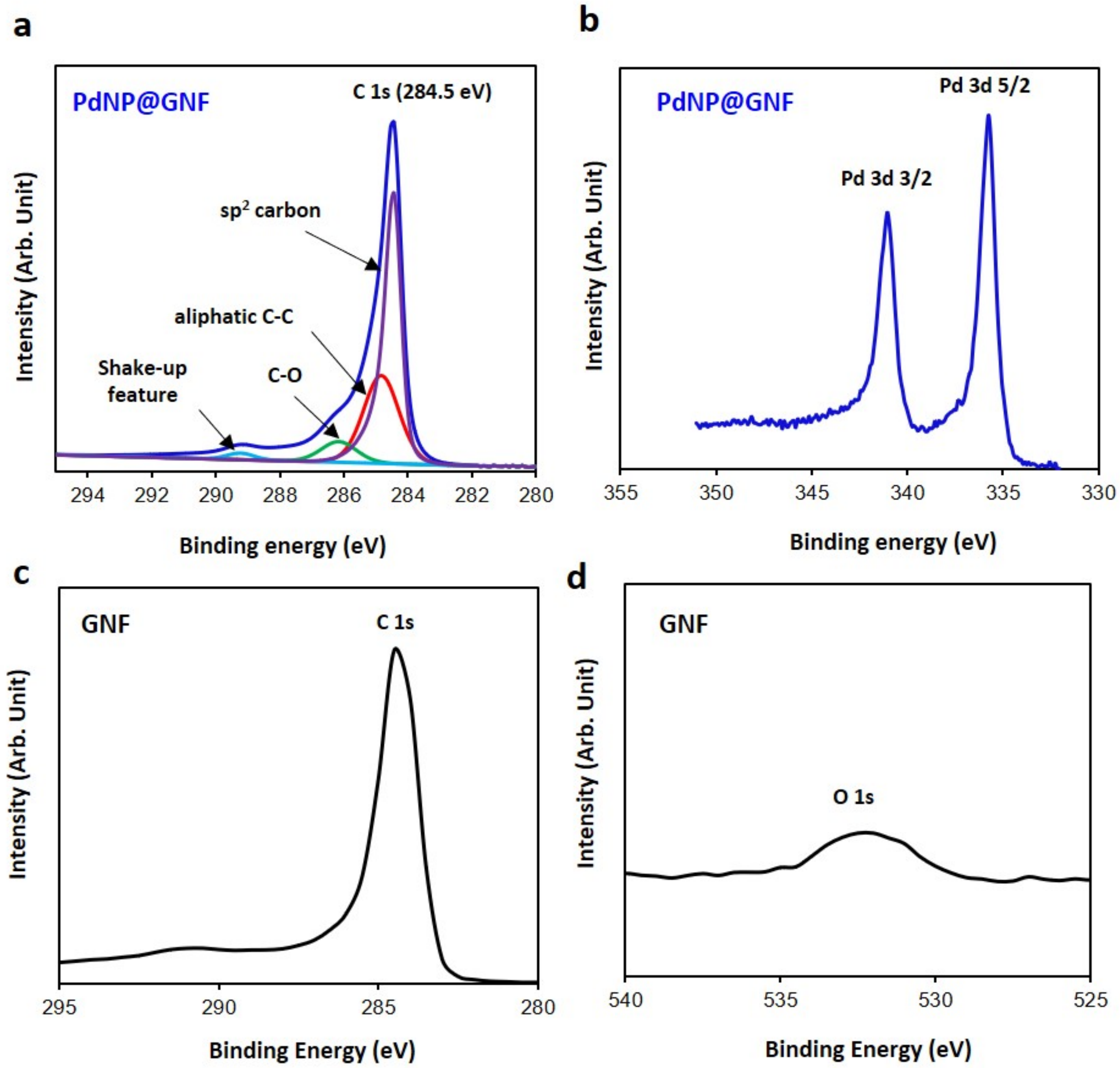

Supplementary Figure 2 | High-resolution XPS spectra in the C_{1s} (**a**) and Pd_{3d} (**b**) regions for PdNP@GNF and in the C_{1s} (**c**) and O_{1s} (**d**) regions for GNF.

Supplementary Table 2 | The surface atomic percentages of relevant elements in shortened GNF and PdNP@GNF calculated from XPS spectra with an error of ± 0.5 %.

	Atomic composition, %			
	**C 1s**	**O 1s**	**Pd 3d**	**Si 2p**
**GNF**	98.1	1.9	–	–
**PdNP@GNF**	82.1	9.7	4.8	3.4

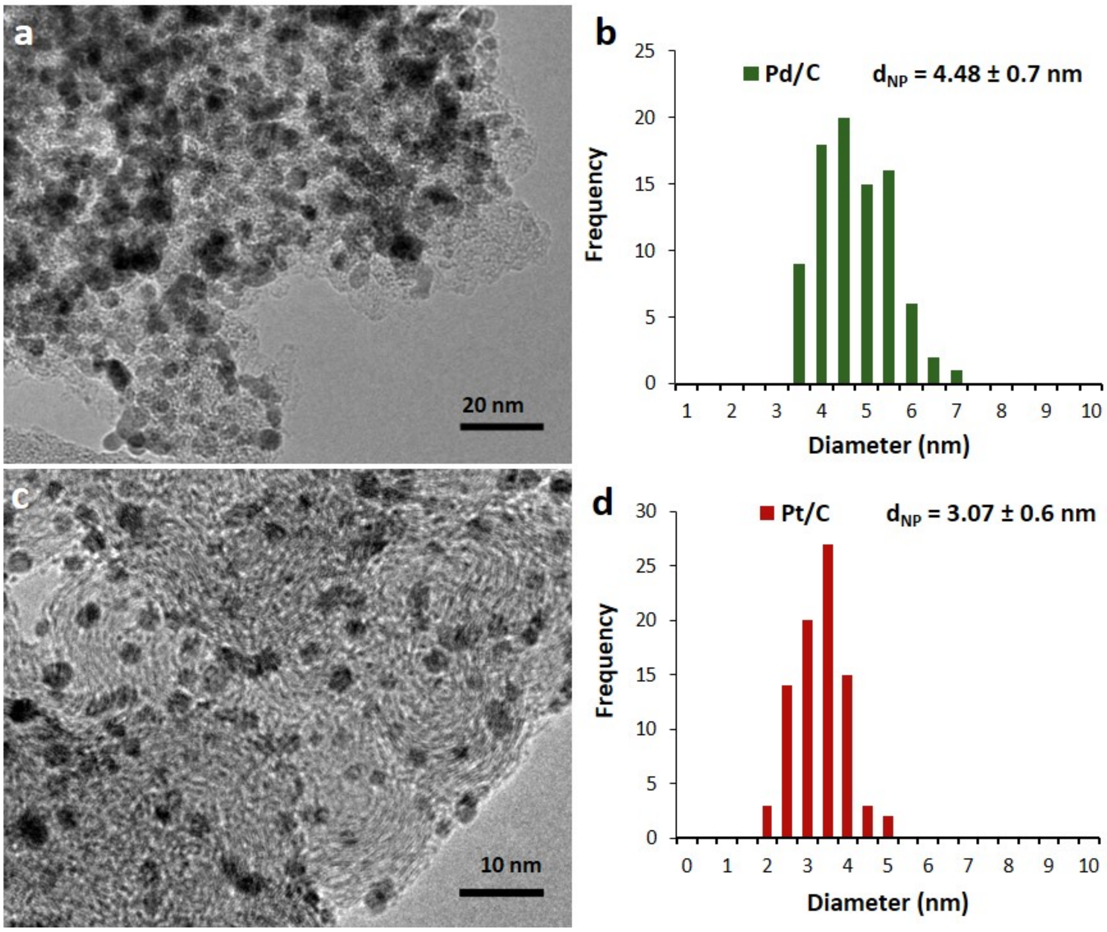

Supplementary Figure 3 | HRTEM images and particle size distributions of Pd/C (**a-b**) and Pt/C (**c-d**) (sizing performed by measuring more than 80 NP per sample).

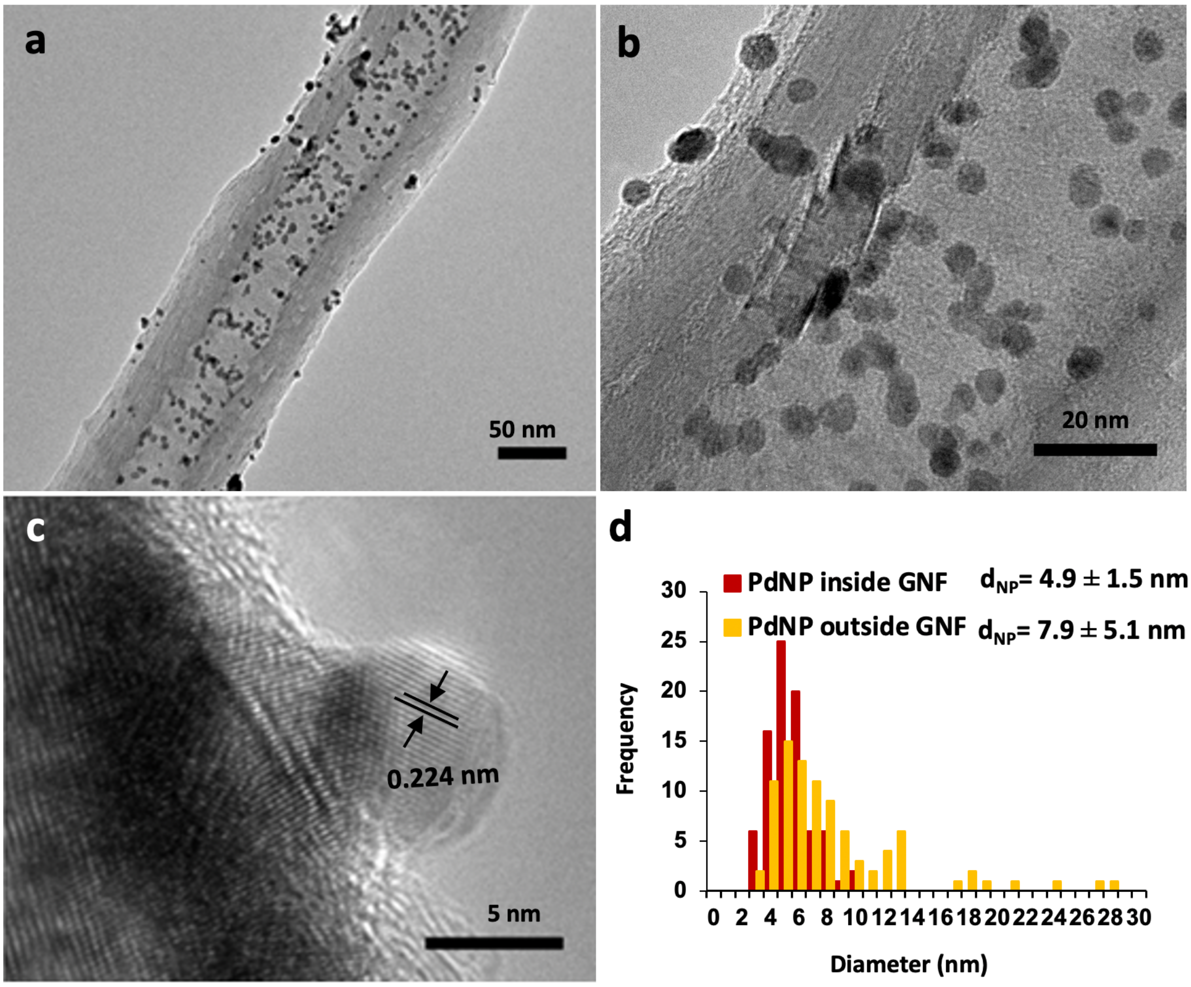

Supplementary Figure 4 | (a-c) HRTEM images of PdNP@GNF after the 5000 cycle durability test, **(c)** PdNP on GNF with a crystallographic interplanar distance of 0.22 nm corresponding to the 2 θ peak centred at 39.5° in the XRD, and **(d)** size distributions of PdNP located at the step-edges within the GNF (PdNP@GNF) and on the external surface of the GNF (PdNP/GNF) (obtained via measuring more than 80 NP in HRTEM images of each sample).

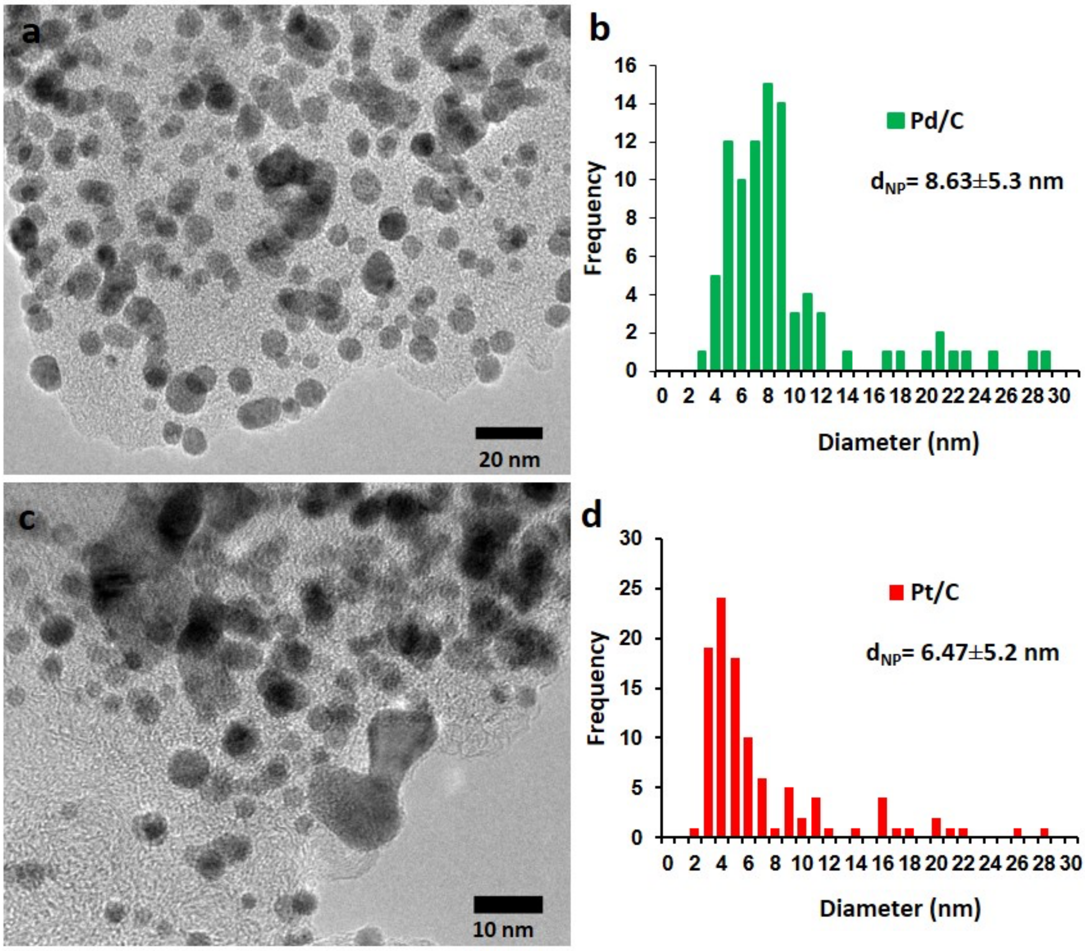

Supplementary Figure 5 | HRTEM images of Pd/C (**a**) and Pt/C (**c**) after stability tests of 5000 cycles and size distributions of Pd/C (**b**) and Pt/C (**d**) obtained through the measurement of more than 80 NP via HRTEM.

Supplementary Table 3 | Onset potential, overpotential, Tafel slopes and exchange current density values for the HER measurements before and after stability tests of 5000 and 30000 cycles for Pd/C, Pt/C and GNF.

	**Onset potential (mV)**	**Overpotential (mV) at -10 mA/ cm^2**	**Tafel Slope (mV /dec)**	**J_0 (mA/cm$^2_{geo}$)**
**Pd/C initial**	-62	76	34	0.058
**Pd/C after 5000 cycles**	-61	67	31.6	0.069
**Pt/C initial**	0	47	28.9	0.983
**Pt/C after 5000 cycles**	0	66	25.7	0.880
**Pt/C after 30000 cycles**	-17	96	71	0.239
**GNF initial**	-320	826	143.5	0.004
**GNF after 5000 cycles**	-57	497	71.1	0.208

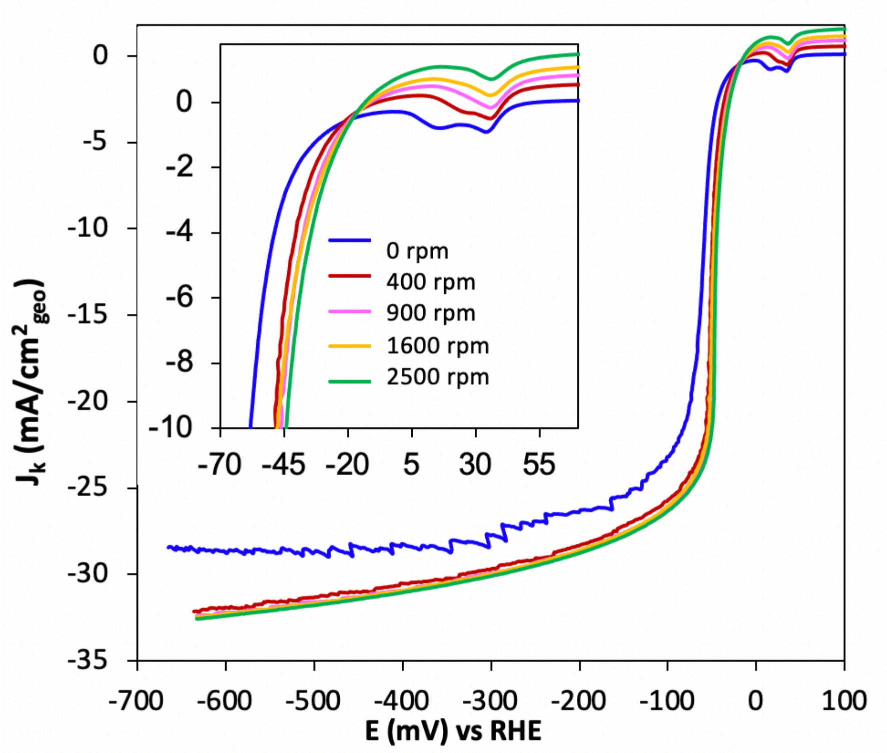

Supplementary Figure 6 | HER polarization curves for the PdNP@GNF electrocatalyst material after 30000 cycles using a carbon counter electrode in H_2-saturated 0.1 M $HClO_4$ without rotation and at different rotation speeds (400, 900, 1600, 2500 rpm) (scanning rate of 10 mV/s). [1]

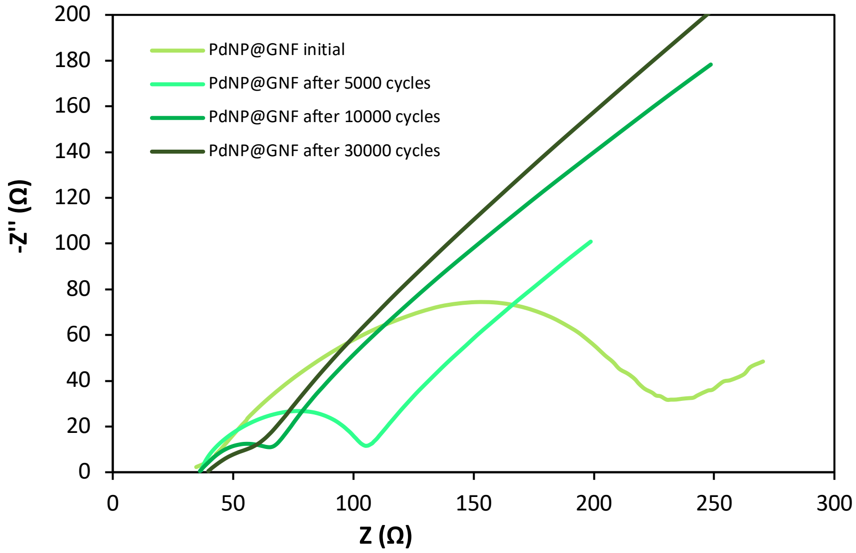

Supplementary Figure 7 | Comparison of the relative change on the electrochemical impedance measure for PdNP@GNF with potential cycling.

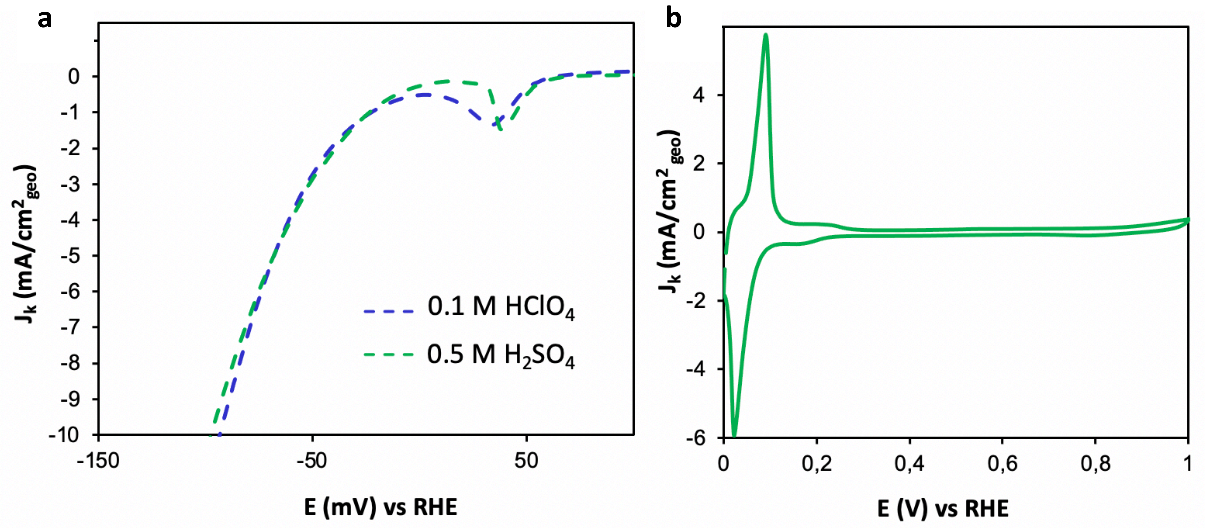

Supplementary Figure 8 | a) Comparison of HER polarization curves for the PdNP@GNF electrocatalyst material after 5000 cycles using carbon rod as the counter electrode in H_2-saturated 0.5 M H_2SO_4 and 0.1 M $HClO_4$. **b)** Cyclic voltammograms of PdNP@GNF after 5000 HER potential cycles at a scan rate of 50 mV/s between 0 V and 1 V in N_2-saturated 0.5 M H_2SO_4.

Supplementary Table 4 | Electrochemical active surface area (ECSA) values in m^2/g_{metal} of PdNP@GNF, Pt/C, Pd/C and GNF with potential cycling.

Catalyst	Initial	5000	10000	30000
PdNP@GNF	11.6±3.6	18.6±1.7	39.5±1.2	39.4±0.8
Pd/C	66.7±2.1	72.7±0.6	--	--
Pt/C	38.7± 0.5	35.7±1.1	--	29.0±1.3

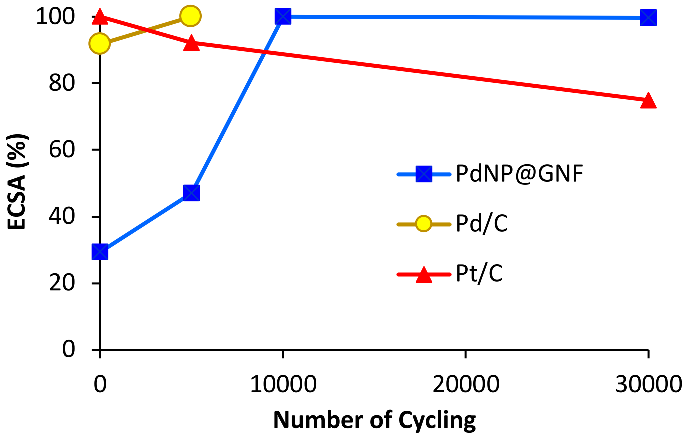

Supplementary Figure 9 | Comparison of the relative change on the electrochemical active surface area (ECSA) values in % of PdNP@GNF, Pt/C, Pd/C and GNF with potential cycling.

Supplementary Table 5 | Specific activity at η = 60 mV values in mA/cm^2 of PdNP@GNF, Pt/C, Pd/C and GNF with potential cycling.

Catalyst	Initial	5000	10000	30000
PdNP@GNF	0.106	0.551	0.580	0.702
Pd/C	0.164	0.154	--	--
Pt/C	0.453	0.497	--	0.339

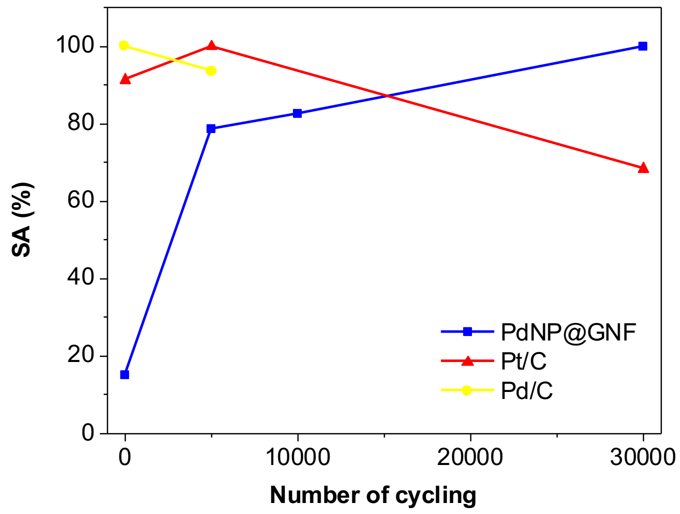

Supplementary Figure 10 | Comparison of the relative change on the specific activity values in % of PdNP@GNF, Pt/C, Pd/C and GNF with potential cycling.

Supplementary Table 6| Mass activity at η = 60 mV values in A/mg_{metal} of PdNP@GNF, Pt/C, Pd/C and GNF with potential cycling.

Catalyst	Initial	5000	10000	30000
PdNP@GNF	0.012	0.102	0.229	0.276
Pd/C	0.109	0.112	--	--
Pt/C	0.176	0.177	--	0.098

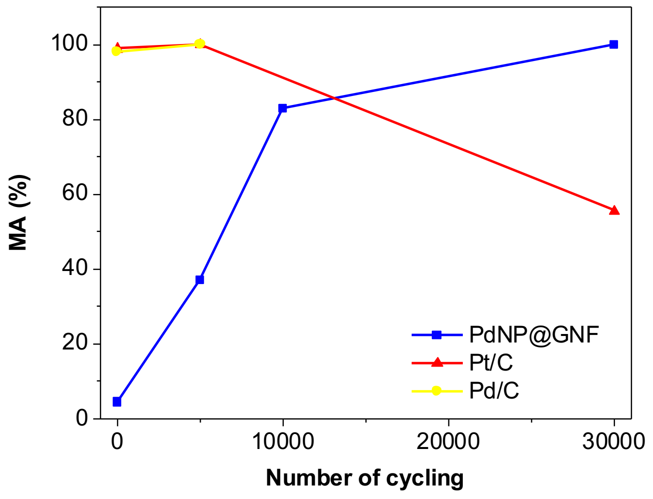

Supplementary Figure 11 | Comparison of the relative change on the mass activity values in % of PdNP@GNF, Pt/C, Pd/C and GNF with potential cycling.

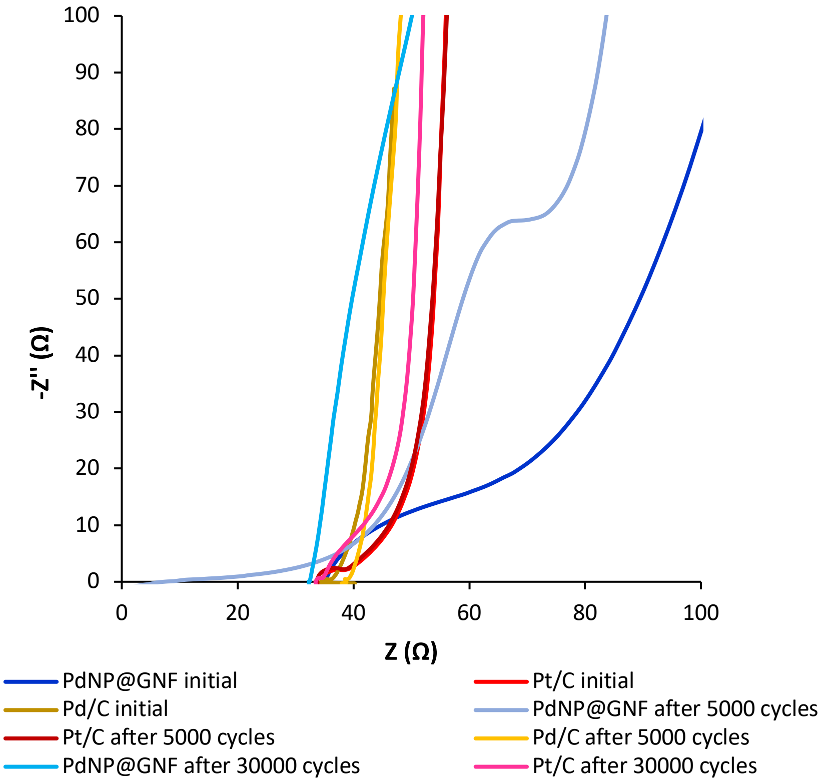

Supplementary Figure 12 | Comparison of the relative change on the electrochemical impedance measure for PdNP@GNF, Pt/C, Pd/C and GNF with potential cycling.

Supplementary Table 7 | Comparison between the electrochemical performances of PdNP@GNF with other recently reported, highly active, HER electrocatalysts.

Catalysts	η (mV) at 10 mA/cm^2	Tafel slope (mV/dec)	j_0 (mA/cm^2)	Electrolyte	η @ 10 mA/cm^2 after certain cycles	References
PdNP@GNF	220	141	0.276	0.1 M $HClO_4$	93 mV at 5000th<br>87 mV at 10000th<br>50 mV at 30000th	**This work**
Pd/HOPG	150	1118.3	N/A	0.1M H_2SO_4	N/A	[2]
CoPd@NC	80	31	N/A	0.5 M H_2SO_4	67 mV at 10000th	[3]
PdCu@PdNCs	68	35	0.74	0.5 M H_2SO_4	~ 70 mV at 5000th	[4]
Ni@Pd/PEI–rGO	90	54	3.91	0.5 M H_2SO_4	~90 mV at 1000th	[5]
PdNi/CNFs (1:2)	55	57	1.536	0.5 M H_2SO_4	~53 mV at 1000th	[6]
Pd/CNFs	101	140	0.678	0.5 M H_2SO_4	N/A	[6]

Pd-MoS_2/MWCNT	120	54	0.071	0.5 M H_2SO_4	~ 125 mV at 500th	[7]
Mo_2C-Pd-9%	28	51	-	0.5 M H_2SO_4	N/A	[8]
Pd–CNx	55	35	0.4	0.5M H_2SO_4	~53 mV at 10000th	[9]
Pd_{16}-CoCNTs	112	56	-	0.5 M H_2SO_4	~ 125 mV at 3000th	[10]
Pd/TiO_2	370 at 1 mA/cm^2	130	NA	phosphate buffer solutions (pH 7.4)	N/A	[11]
fMWCNTs@Pd/TiO_2	170 at 1 mA/cm^2	130	0.06	phosphate buffer solutions (pH 7.4)	N/A	[11]

* Estimated from the HER polarization curves of the data reported in related reference.

All potentials are reported *vs* RHE.

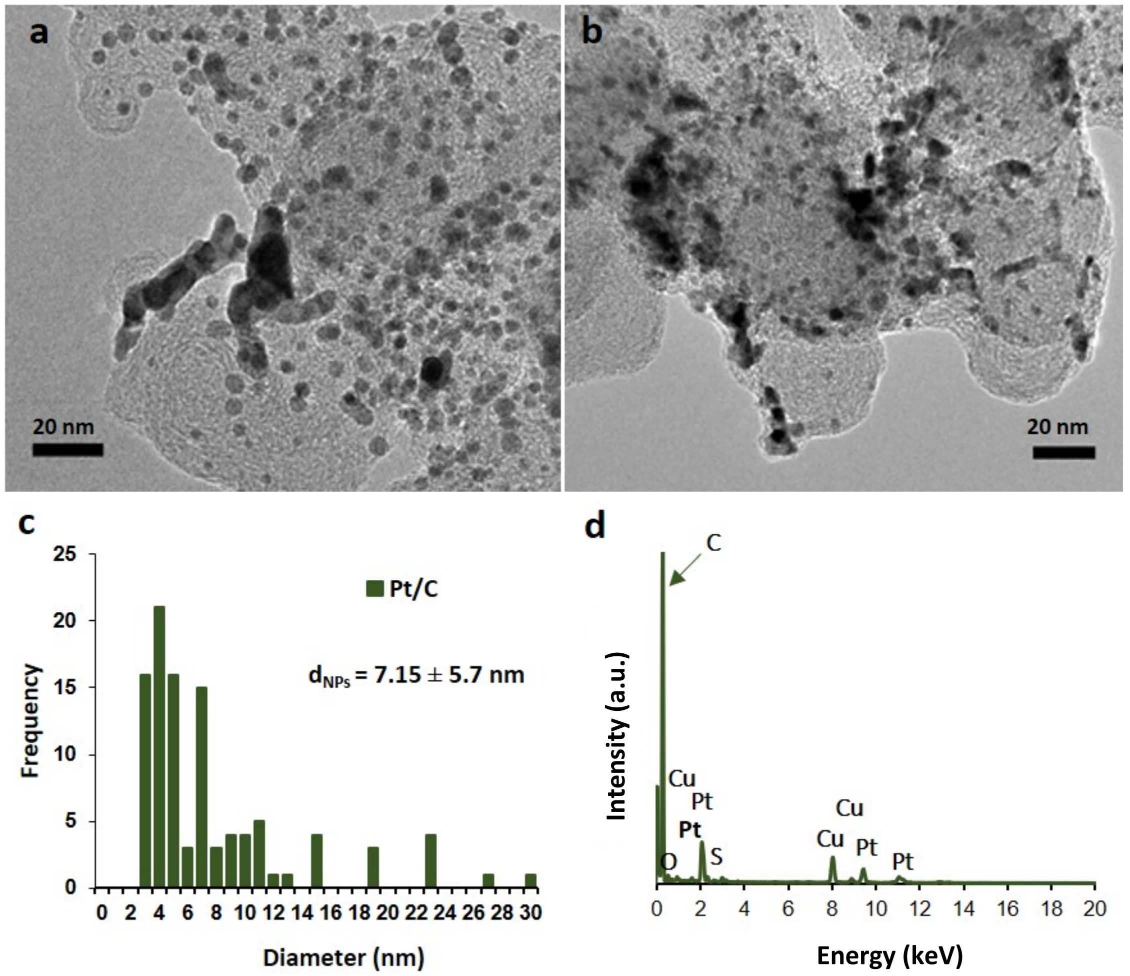

Supplementary Figure 13| HRTEM images of Pt/C after 30000 cycles (**a-b**), the particle size distribution of Pt NP (measured for more than 80 NP) on Pt/C (**c**) and EDX analysis (**d**).

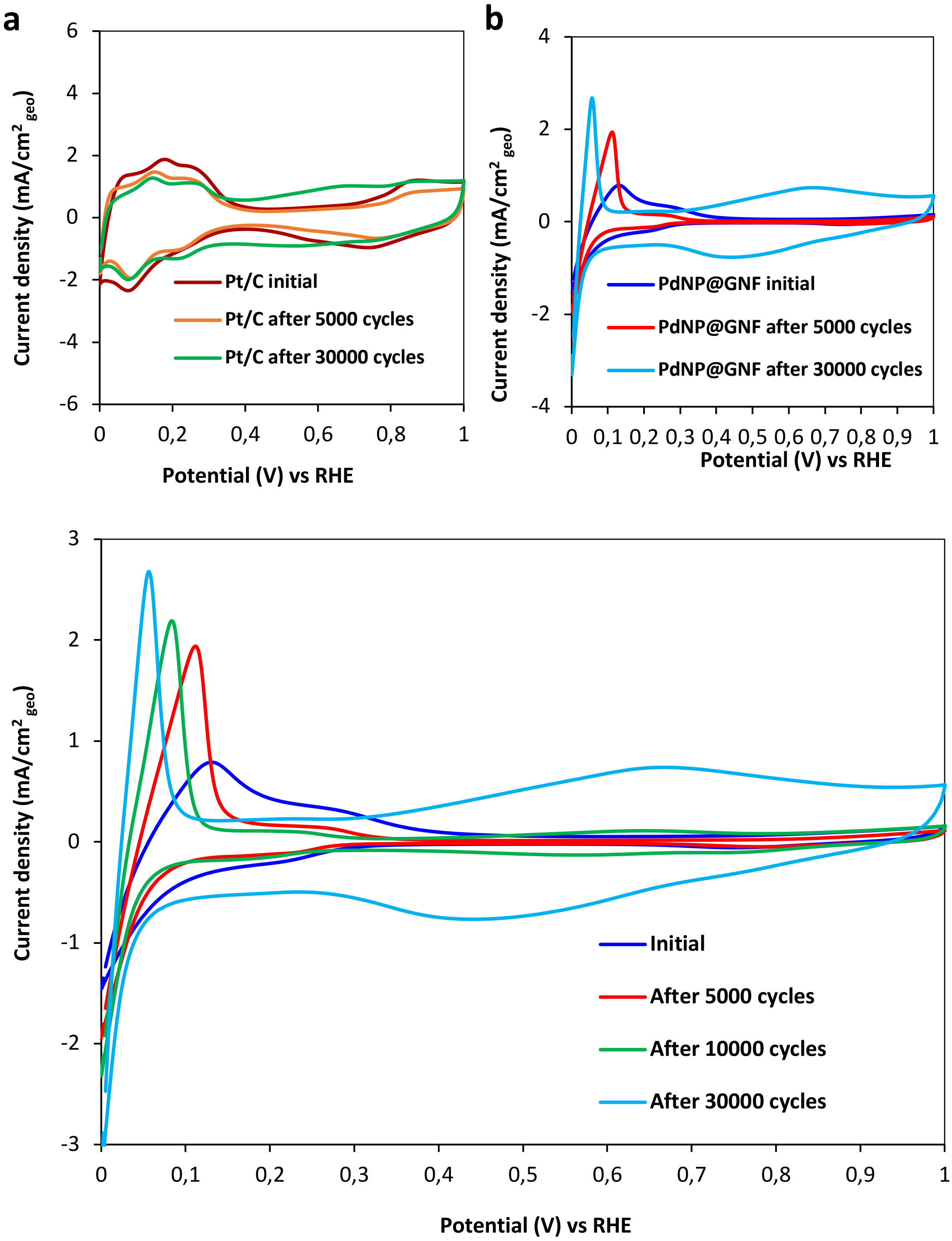

Supplementary Figure 14| Cyclic voltammograms of Pt/C (**a**) and PdNP@GNF (**b-c**) as a function of cycle number at a scan rate of 50 mV/s between 0 V and 1 V in N_2 saturated 0.1 M $HClO_4$. (Currents were normalized by the geometric electrode surface area of the GC electrode). Please note that the peak rising at Hupd desorption region between 0-1.15 V with increasing HER stability cycling is due to the absorption of protons to the opened step-edges.

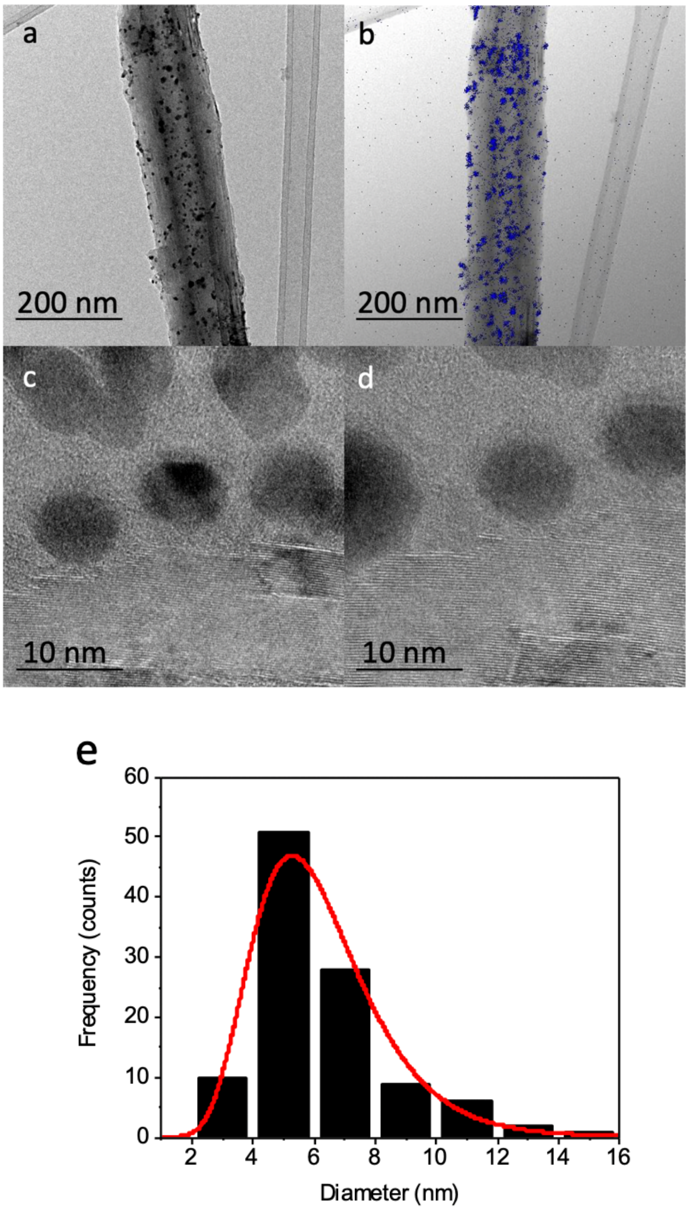

Supplementary Figure 15 | (**a**) HRTEM and (**b**) STEM-EDX mapping of PdNP@GNF after 30000 HER potential cycling using carbon rod counter electrode. Regions in blue shows the distribution of Pd. (**c**) and (**d**) figures show high HRTEM magnification of the sample where Pd nanoparticles with an average size of 5.2 ± 1.7 nm appears to be wired to opened step edges. (**e**) Size distribution obtained via measuring more than 100 NP in HRTEM images.

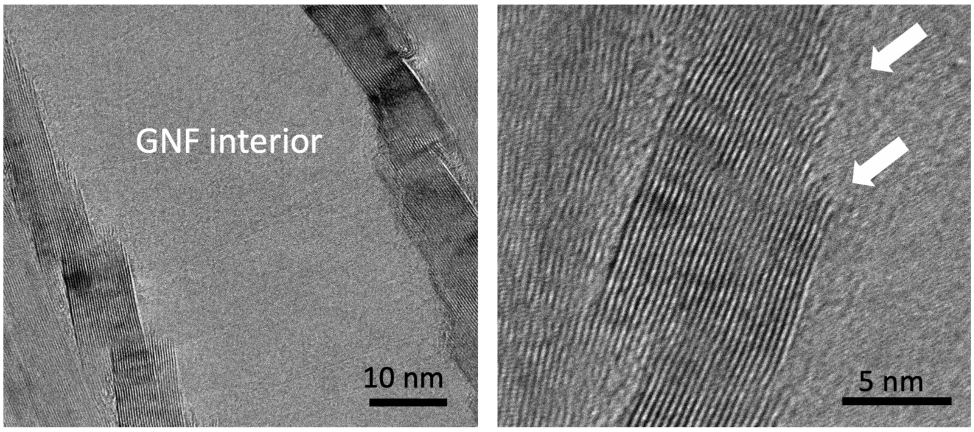

Supplementary Figure 16 | HRTEM of a GNF after annealing at 600 °C for 5 hours in air to selectively open the step-edges (a close visualization of an open the step-edge on the right).

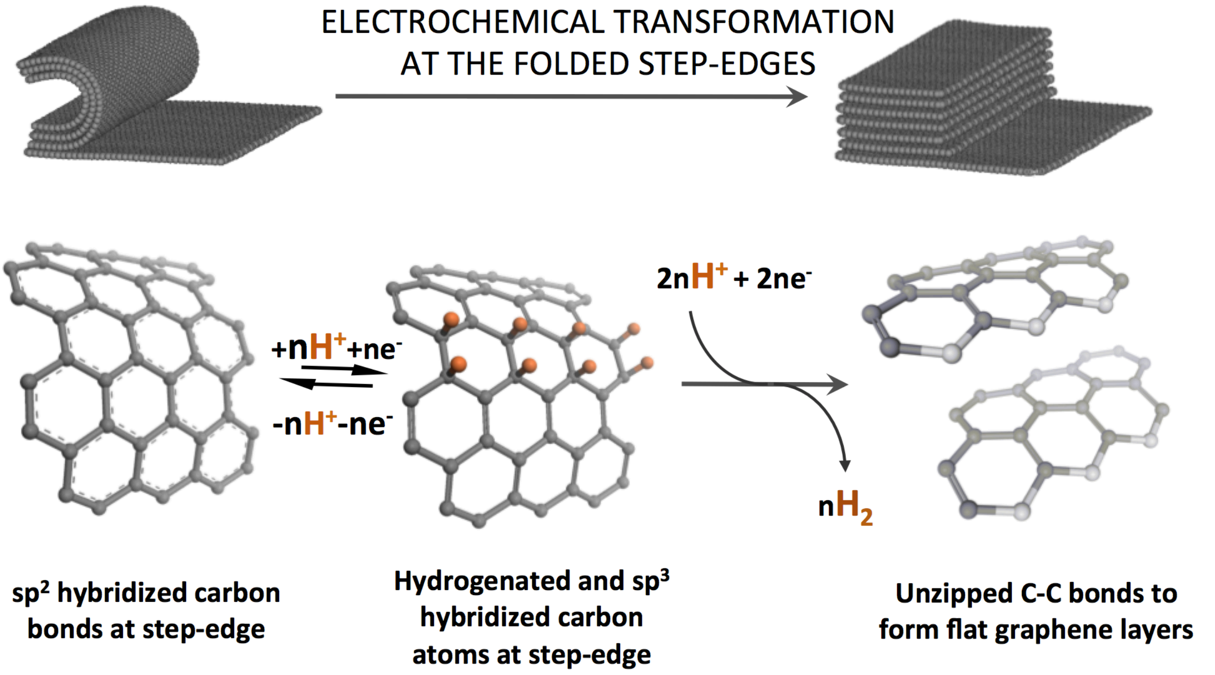

Supplementary Figure 17 | Proposed mechanism showing how under HER conditions protons may react with the C=C bonds at the curved graphitic step-edge along the zigzag direction of the carbon sheet. Initially they reversibly form a zigzag line of C-H bonds, and then, further hydrogenation leads to the rupture of the curved carbon sheet (carbon dangling bonds, light grey) and the formation of flat graphene layers with hydrogen terminating carbon bonds in acid media.

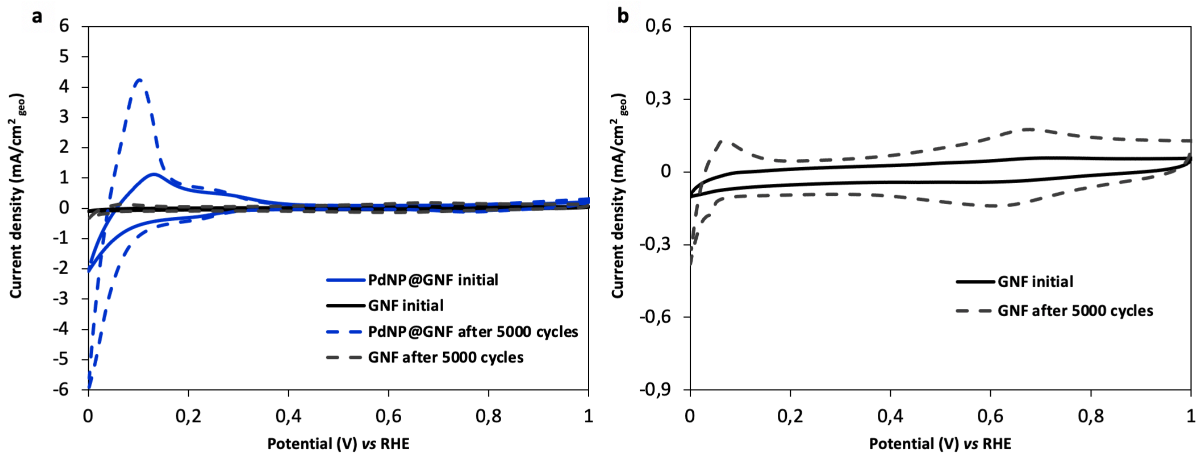

Supplementary Figure 18| Cyclic voltagrams at scan rates of 50 mV/s between 0 V and 1 V in N_2 saturated 0.1 M $HClO_4$ of GNF and PdNP@GNF as deposited initially and after the stability tests of 5000 and 30000 cycles of HER. (Currents were normalized by the geometric electrode surface area of the GC electrode).

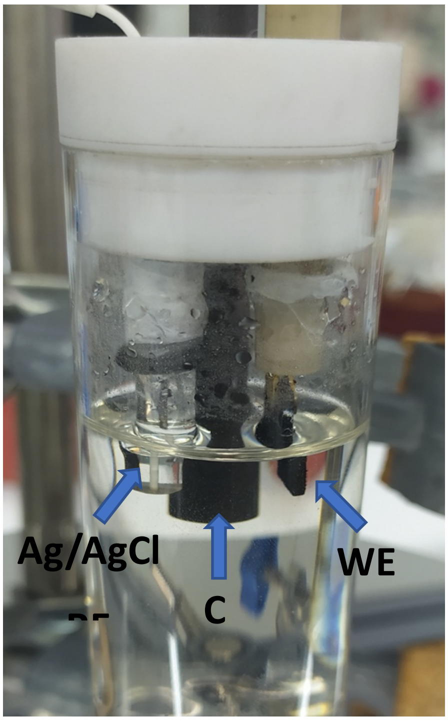

Supplementary Figure 19| The three set-up electrochemical cell used for the 24 hours chronoamperometry experiment. Carbon rod was used as counter electrode and Ag/AgCl as reference electrode. The electrolyte was 0.1 M $HClO_4$.

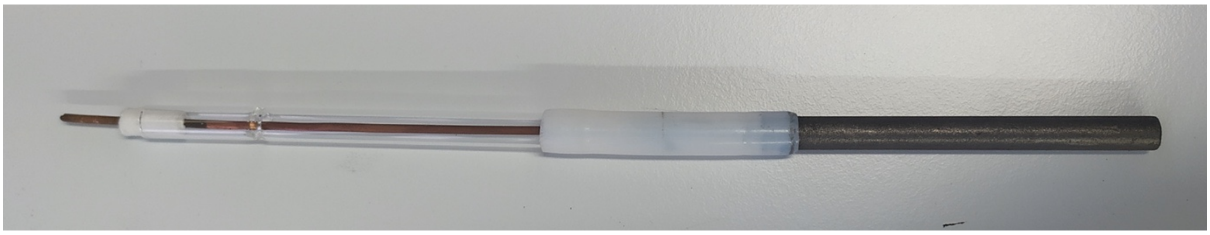

Supplementary Figure 20 | Carbon rod counter electrode used for electrochemical measurements.

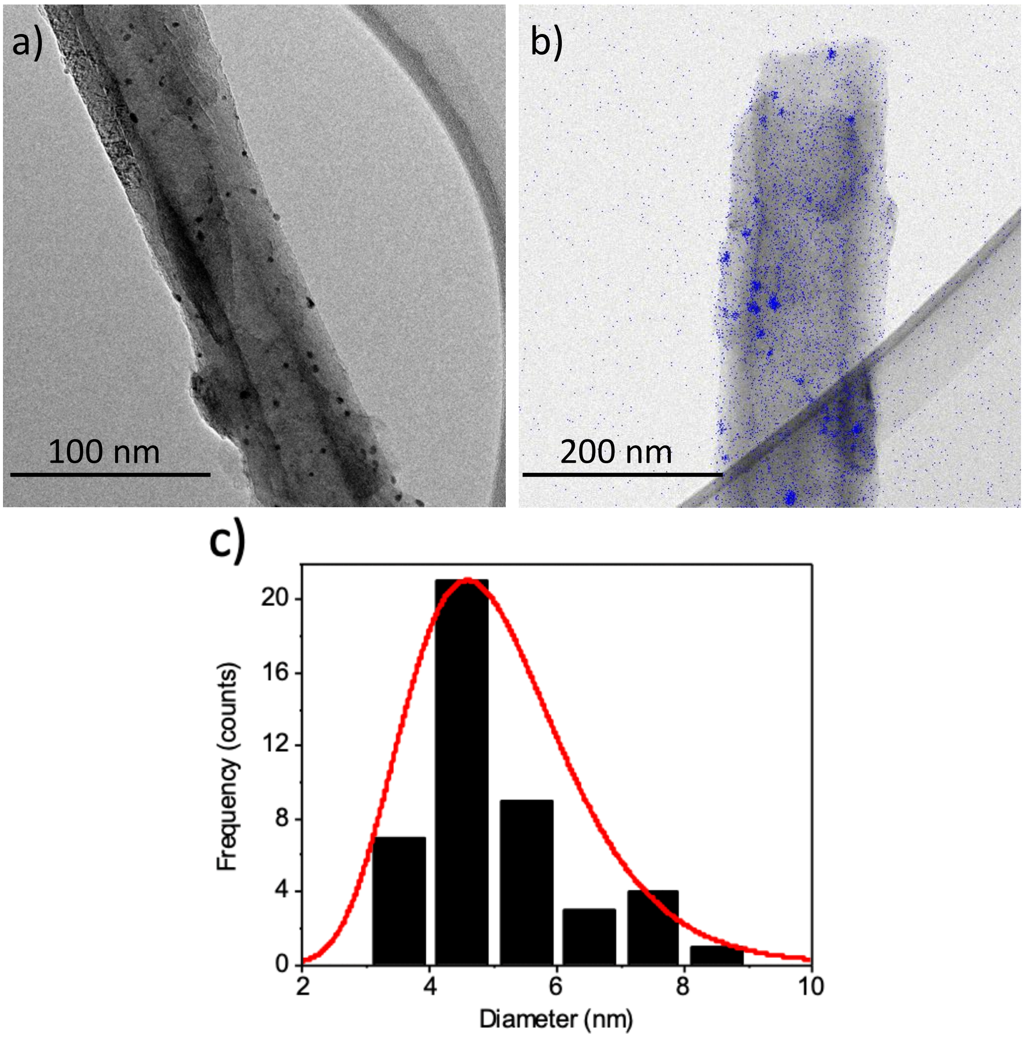

Supplementary Figure 21 | (**a**) HRTEM and (**b**) STEM-EDX mapping of PdNP@GNF after 24 hours chronoamperometry experiments. Pd in blue. **(c**) size distributions of PdNP located at the step-edges within the GNF (PdNP@GNF) (obtained via measuring more than 50 NP in HRTEM images).

References

[1] Zheng, J., Yan, Y., Xu, B. Correcting the Hydrogen Diffusion Limitation in Rotating Disk Electrode Measurements of Hydrogen Evolution Reaction Kinetics. Journal of The Electrochemical Society, 162 (14) F1470-F1481 (2015))

[2] Ju, W. et al. Palladium Nanoparticles Supported on Highly Oriented Pyrolytic Graphite: Preparation, Reactivity and Stability, *ChemElectroChem*, **2**, 547−558 (2015).

[3] Chen, J. et al. Active and Durable Hydrogen Evolution Reaction Catalyst Derived from Pd-Doped Metal–Organic Frameworks, *ACS Appl. Mater. Interfaces,* **8**, 13378−13383 (2016)**.**

[4] Li, J. et al. PdCu@Pd Nanocube with Pt-like Activity for Hydrogen Evolution Reaction, *ACS Appl. Mater. Interfaces,* **9**, 8151−8160 (2017).

[5] J. Li, et al. Ni@Pd/PEI–rGO stack structures with controllable Pd shell thickness as advanced electrodes for efficient hydrogen evolution, *J. Mater. Chem. A,* **3**, 11261-11268 (2015).

[6] Chen, J. et al. Carbon nanofiber-supported PdNi alloy nanoparticles as highly efficient bifunctional catalysts for hydrogen and oxygen evolution reactions, *Electrochim. Acta*, **246**, 17–26 (2017).

[7] Heydari-Bafrooei E. & Askari, S. Electrocatalytic activity of MWCNT supported Pd nanoparticles and MoS_2 nanoflowers for hydrogen evolution from acidic media, *Int. J. of Hydrogen Energy*, **42**, 2961-2969 (2017).

[8] T. Li, et al. Palladium nanoparticles grown on β-Mo_2C nanotubes as dual functional electrocatalysts for both oxygen reduction reaction and hydrogen evolution reaction, *Int. J. of Hydrogen Energy*, **43**, 4932 -4941 (2018).

[9] Bhowmik, T. et al. Palladium Nanoparticle–Graphitic Carbon Nitride Porous Synergistic Catalyst for Hydrogen Evolution/Oxidation Reactions over a Broad Range of pH and Correlation of Its Catalytic Activity with Measured Hydrogen Binding Energy, *ACS Catal.*, **6**, 1929−1941 (2016).

[10] Huang, B. et al. Paragenesis of Palladium–Cobalt Nanoparticle in Nitrogen-Rich Carbon Nanotubes as a Bifunctional Electrocatalyst for HydrogenEvolution Reaction and Oxygen-Reduction Reaction, *Chem. Eur. J.,* **23** , 7710-7718 (2017).

[11] Valenti, G. et al. Co-axial heterostructures integrating palladium/titanium dioxide with carbon nanotubes for efficient electrocatalytic hydrogen evolution, *Nat.Commun.,* **7**, 13549 (2016).
